# DisC^2^o-HD: Distributed causal inference with covariates shift for analyzing real-world high-dimensional data

**Published:** 2025

**Authors:** Jiayi Tong, Jie Hu, George Hripcsak, Yang Ning, Yong Chen

**Affiliations:** Department of Biostatistics, Epidemiology and Informatics, University of Pennsylvania, Philadelphia, PA 19104, USA; Department of Biostatistics, Epidemiology and Informatics, University of Pennsylvania, Philadelphia, PA 19104, USA; Department of Biomedical Informatics, Columbia University, New York, NY 10027, USA; Department of Statistics and Data Sciences, Cornell University, Ithaca, NY 14853, USA; Department of Biostatistics, Epidemiology and Informatics, University of Pennsylvania, Philadelphia, PA 19104, USA

**Keywords:** Causal Inference, Distribution Shift, Federated Learning, High-dimensional Data, Real-World Data

## Abstract

High-dimensional healthcare data, such as electronic health records (EHR) data and claims data, present two primary challenges due to the large number of variables and the need to consolidate data from multiple clinical sites. The third key challenge is the potential existence of heterogeneity in terms of covariate shift. In this paper, we propose a distributed learning algorithm accounting for covariate shift to estimate the average treatment effect (ATE) for high-dimensional data, named DisC^2^o-HD. Leveraging the surrogate likelihood method, our method calibrates the estimates of the propensity score and outcome models to approximately attain the desired covariate balancing property, while accounting for the covariate shift across multiple clinical sites. We show that our distributed covariate balancing propensity score estimator can approximate the pooled estimator, which is obtained by pooling the data from multiple sites together. The proposed estimator remains consistent if either the propensity score model or the outcome regression model is correctly specified. The semiparametric efficiency bound is achieved when both the propensity score and the outcome models are correctly specified. We conduct simulation studies to demonstrate the performance of the proposed algorithm; additionally, we apply the algorithm to a real-world data set to present the readiness of implementation and validity.

## 1 Introduction

Causal inference, which aims to elucidate the cause-effect relationships underlying the observed phenomena, usually relies on carefully designed experiments to establish causality (Hernán and Robins, 2010). However, in many domains, conducting controlled experiments may be unfeasible, leaving researchers to look for alternative methods. The increasing amount of real-world data (RWD) that captured the patients’ clinical information offer a valuable opportunity for the researchers to investigate the causal relationships on a larger scale. By providing resourceful and rich observational data, the RWD shed light on building complex healthcare systems, inform evidence-based decision making, and drive advancements across diverse fields in addition to public health such as social sciences, economics, and beyond.

In the past few decades, the distributed research networks (DRNs) have been built to facilitate large-scale observational studies, covering large sample sizes and diverse populations, for example, the Observational Health Data Sciences and Informatics (OHDSI) consortium (Hripcsak et al., 2015), an international network of researchers and observation health databases, and the Patient-Centered Clinical Research Network (PCORnet) (Fleurence et al., 2014; Collins et al., 2014), which covers groups of diverse healthcare institutions and CRNs across the U.S. These research networks are highly valuable for clinical research by improving statistical power and enhancing the generalizability of the findings (Friedman et al., 2010). The growth of research networks has made it possible to analyze rare events and improve the accuracy of statistical models.

However, when utilizing large-scale RWD collected from CRNs for causal inference, there are three critical challenges to address. The first challenge revolves around the difficulty of sharing patient-level data, often due to privacy concerns and varying policy regulations in biomedical research (Behlen and Johnson, 1999). Sharing individual patient-level data can be time-consuming, logistically challenging, or infeasible in practice. The second major challenge arises when interest lies in conducting comparative effectiveness research via causal inference using RWD, where high-dimensional covariates collected in RWD are used to control the impact of confounders. Last but not least, the existence of population heterogeneity, also known as distribution shift or covariate shift, is also a key challenge to consider in practice. The differences in the underlying population could be caused by factors such as geographical variability in disease patterns, variations in patient characteristics, and regional differences in practice patterns. For example, there are studies using multiple electronic health records (EHR) datasets from Mayo Clinics and Vanderbilt University Medical Center (VUMC) to investigate the causal effects of candidate non-cancer drugs to be used for the treatment of cancer for drug repurposing (Xu et al., 2015; Wu et al., 2019). These studies successfully identified potential candidates for antineoplastic repurposing. A notable observation in these studies is that patient characteristics, including factors such as racial distribution and medication usage (such as insulin utilization), exhibit variations across the different sites. When conducting multi-site analyses in which patient-level data cannot be shared, it is essential to employ statistical methods that account for covariate shifts. Ignoring these differences can lead to biased estimates of causal relationships, an increased risk of overfitting, compromised generalizability of the findings, and potentially ineffective decision-making.

To address the first challenge in data sharing, a divide-and-conquer procedure is commonly used (Zhang et al., 2013; Lee et al., 2017; Battey et al., 2018). In particular, Battey et al. (2018) is one of the earliest innovations on distributed hypothesis testing for divide-and-concur estimator with high-dimensional data. After calculating and sharing the local estimators from the local patient-level data at each data site to the lead site or coordinating center, the final estimator is obtained by taking the average over the local estimators. Though sharing the estimators across sites mitigates the need in sharing patient-level data, the theoretical and empirical performance of this simple average method is suboptimal, especially when dealing with a large number of clinical sites and rare disease setting in multi-site studies (Duan et al., 2020b). In the past few years, an enhanced distributed learning, known as the surrogate likelihood approach, was proposed for association studies and prediction tasks (Wang et al., 2017; Jordan et al., 2018; Duan et al., 2018, 2020a,b, 2022). By requiring summary statistics from collaborating sites, the method is communication-efficient and privacy-preserving. In real-world settings, communication costs present a significant challenge, particularly in collaborative studies where transferring summary-level statistics demands considerable human labor. In response, it is essential to develop a communication-efficient distributed learning algorithm that minimizes communication rounds across sites.

In the context of addressing the second challenge posed by high-dimensional settings using RWD, considerable efforts have been dedicated to estimating the average treatment effect (ATE) in recent years. A number of notable methods have emerged, each of which presents innovative strategies. Belloni et al. (2014), Farrell (2015), Belloni et al. (2017), and Chernozhukov et al. (2018), have proposed a two-step approach. In this approach, they advocate first estimating the propensity score through penalized maximum likelihood and subsequently utilizing the efficient score function to estimate the ATE. Athey et al. (2018) introduced a different perspective by proposing approximate residual balancing. Notably, this approach eliminates the need for a propensity score model, while maintaining a requirement for linearity in covariates for the outcome model. This method was shown to be semiparametrically efficient and the balancing weights converge to the inverse propensity score but with a slower rate under suitable regularity conditions (Hirshberg and Wager, 2021). Bradic et al. (2019) contributed an estimator that excels in situations of rate/sparsity double robustness. The key advantage of this estimator is its root-n consistency even when either the propensity score model or the outcome model lacks sparsity, as long as the other model exhibits sufficient sparsity. Additionally, Tan (2020a) and Ning et al. (2020) proposed the high-dimensional covariate balancing propensity score method, which provides doubly robust confidence intervals for ATE involving high-dimensional covariates. These methods yield root-n consistent and asymptotically normal estimators, contingent on the accurate specification of either the propensity score model or the outcome model.

However, it is important to note that these methods are not specifically designed to accommodate scenarios where data are distributed across multiple clinical sites within research networks. Recent studies in this direction have led to the proposal of distributed learning algorithms for ATE estimation in causal inference (Xiong et al., 2021; Han et al., 2021), where the propensity score model and outcome model were used for causal effect estimation. Nevertheless, these methods cannot be applied directly to analyze high-dimensional data, as lasso-type estimators may not be aggregated directly in multisite settings (Battey et al., 2018).

In this paper, we propose a solution called DisC^2^o-HD to simultaneously address all three challenges: data sharing, distributed causal inference for high-dimensional data, and covariate shift. Our method is specifically designed for analyzing real-world high-dimensional data and incorporates three key features:

- Firstly, DisC^2^o-HD leverages the surrogate method to estimate the propensity score model and outcome model. Our proposed method can be implemented within a few rounds of communication, requiring only a single round of communication for participating sites to transfer summary-level statistics when fitting each model; all estimation iterations are conducted within a single local site or a designated lead site. This efficiency feature makes the proposed method more applicable to practical settings, enabling the generation of real-world data-based evidence.
- Secondly, our method effectively handles high-dimensional data to estimate average treatment effects (ATE). By properly integrating the rich information contained in high-dimensional data into our inferential procedure, we obtain more reliable estimates of causal effects. Furthermore, our method demonstrates robustness to model misspecifications. Even if either the propensity score model or the outcome model is incorrectly specified, our distributed algorithm produces results comparable to the gold standard method that relies on pooling patient-level data.
- Thirdly, DisC^2^*o*-HD accounts for heterogeneous populations (e.g., differences in population or systematic variations) across multiple sites. It is capable of accommodating variations among different sites, ensuring the applicability of our method to diverse patient cohorts. Its robustness in handling the distributional differences makes it highly applicable in real-world situations.

Overall, DisC^2^o-HD addresses the challenges of data sharing, distributed causal inference for high-dimensional data, and covariate shift. It offers a comprehensive and reliable approach for analyzing real-world high-dimensional data while protecting privacy and accommodating population heterogeneity across multiple sites.

We use the following notation. For $v=\left(v_{1},\ldots ,v_{p}\right)\in R^{p}$ and $1⩽q⩽\infty ,\;\|v{\|}_{0}=|supp(v)|$, where $supp(v)=\left\{j:v_{j}\neq 0\right\}$, and $\|v{\|}_{q}={\left(\sum_{i=1}^{d}{\left|v_{i}\right|}^{q}\right)}^{1/q}$. The Orlicz norm associated with a Young’s modulus ψ of X is defined by $\left\|X{\|}_{\psi }=inf\{C>0:E[\psi (|X|/C)]⩽1\right\}$. $\lambda_{min}(A)$ and $\lambda_{max}(A)$ represent the minimal and maximal eigenvalues of A if we have a symmetric matrix A. For two positive sequences $a_{n}$ and $b_{n},\;a_{n}≍b_{n}$ if there exist $C,\;C^{\prime }>0$ such that $C⩽a_{n}/b_{n}⩽C^{\prime }$ holds. For $\psi_{1}=e^{x^{2}}-1$, a random variable X is sub-Gaussian, if ${\left\|X\right\|}_{\psi_{1}}<\infty$. Denote a∨b=max(a,b).

## 2 Methodology

Given the context of analyzing multisite data from research networks, we assume that individual patient data (IPD) are stored at K sites in a distributed manner and IPD cannot be shared across sites. Without loss of generality and for notation simplicity, we assume that each site has equal sample size n. We denote N as the total sample size of the pooled data from all K clinical sites (i.e., N=Kn). For the i-th patient from the k-th site, we observe ($T_{ki},Y_{ki},X_{ki}$), where $T_{ki}\in \{0,\;1\}$ is the binary treatment assignment, $Y_{ki}$ is the outcome variable, and $X_{ki}$ is a p-dimensional vector of pre-treatment covariates.

**Figure 1:**
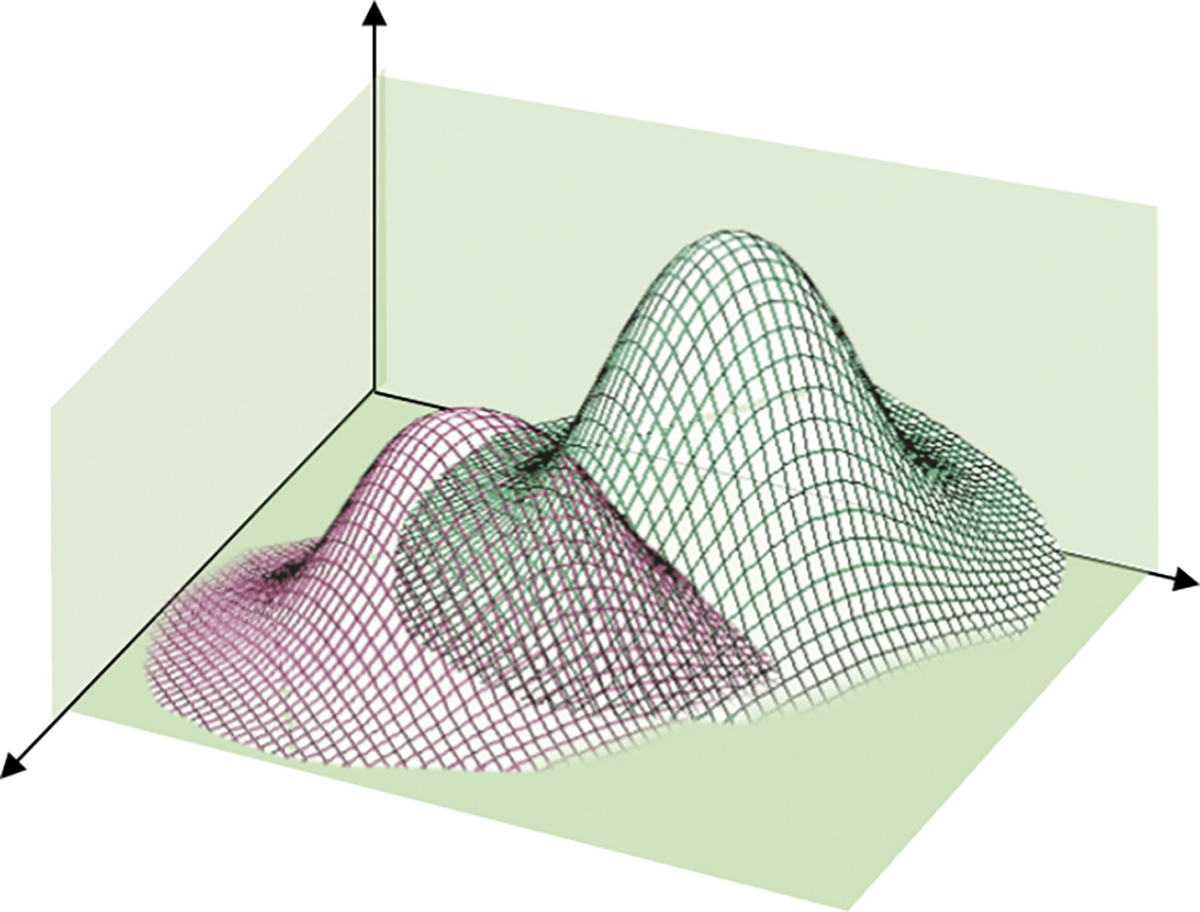
Illustration of distribution shift in 3-dimension plot, where mean values of the densities are the same, but the values of covariance matrices are different.

We formulate the problem by considering the existence of covariate shifts (i.e., heterogeneous covariate distributions) across the sites. The term “covariate shift” refers to the variation in the distribution of the covariates across distinct sites. To provide a concrete illustration, suppose we have a single covariate, denoted as X, which is accessible across all sites. This covariate X follows a normal distribution at each site. The “covariate shift” scenario of X means the distribution of X at site A might have different mean and variance values compared to the distribution of X at another site. The definition of distribution shift is illustrated visually in Figure 1 and defined as follows:

### Definition 1

Let’s denote by P and Q two probability measures associated with random variables X and $X^{\prime }$ respectively, defined on the same probability space (Ω,F), and suppose they admit density functions f and g. If there exists a set A such that for all x in A,f(x)≠g(x) and the Lebesgue measure of A (denoted by λ(A)) is not zero, then the density functions f and g (and thus the distributions P and Q) are different. This can be written formally as:

∃A∈F,λ(A)>0suchthat∀x∈A,f(x)≠g(x)

This essentially means that there is a non-negligible set of outcomes where the two random variables X and $X^{\prime }$ have different densities.

The potential outcome under treatment and the potential outcome under control for the k-th site are denoted as $Y_{ki}(1)$ and $Y_{ki}(0)$, respectively. The observed outcome $Y_{ki}$ is denoted as $Y_{ki}=T_{ki}Y_{ki}(1)+\left(1-T_{ki}\right)Y_{ki}(0)$. The parameter of interest by taking the covariate shift into account is the average treatment effect (ATE) over the K populations, defined as

$$\Delta^{*}=\frac{1}{K}\sum_{k=1}^{K}E\left[Y_{ki}(1)-Y_{ki}(0)\right]=\frac{1}{K}\sum_{k=1}^{K}E\left[Y_{ki}(1)\right]-\frac{1}{K}\sum_{k=1}^{K}E\left[Y_{ki}(0)\right].$$

The terms $\tau_{1}^{*}\;:=\frac{1}{K}\sum_{k=1}^{K}E\left[Y_{ki}(1)\right]$ and $\tau_{0}^{*}\;:=\frac{1}{K}\sum_{k=1}^{K}E\left[Y_{ki}(0)\right]$ can be obtained in similar way by replacing the treatment assignment. Therefore, in the following subsections including derivations, equations, and algorithms, we will focus on the estimation of $\tau_{1}^{*}$, which is the expected outcome for the treated cohort.

At k-th site, we consider the following logistic propensity score (PS) model and a linear outcome model (OM):

(1) $$P\left(T_{ki}=1|X_{ki}\right)=\pi \left(X_{ki}^{T}\theta \right),$$

(2) $$E\left[Y_{ki}(1)|X_{ki}\right]=X_{ki}^{T}\beta ,$$

where θ is a p-dimensional unknown vector that is homogeneous across all sites, π(z)=1/(1+exp(-z)), and β is a p-dimensional unknown vector. We assume that the PS model is homogeneous across all sites, and the same applies to the OM model. In this paper, we allow the models (1) and (2) to be misspecified. In the following sections, we will first present the algorithm and theoretical results by assuming both models are correctly specified. Then, we will further present the asymptotic distribution of the proposed estimator when either model is mis-specified in Section 3.4.

### 2.1 Background: Pooled method

If all of the patient-level data from K sites can be pooled together, two potential challenges exist: high-dimensional data and covariate shift. To address the first challenge, a variety of methods have been developed, as reviewed in the Introduction. To fix the idea, in this work we focus on the high-dimension covariate balancing method proposed by Ning et al. (2020). The first step is to estimate the propensity score via the following $\ell_{1}$-penalized estimator

(3) $${\overset{ˆ}{\theta }}_{\text{pooled}}=\underset{\theta \in R^{p}}{\arg min}Q_{N}(\theta )+\lambda_{\text{pooled}}^{\prime }{\left\|\theta \right\|}_{1},$$

where $Q_{N}(\theta )=\frac{1}{K}\sum_{k=1}^{K}Q_{k}(\theta )$ with

(4) $$Q_{k}(\theta )=\frac{1}{n}\sum_{i=1}^{n}\left\{\left(1-T_{ki}\right)\;X_{ki}^{T}\theta +T_{ki}/exp\left(X_{ki}^{T}\theta \right)\right\}$$

with $Q_{k}(\theta )$ being a generalized quasilikelihood function from the k th site, which is similar to the quasi-likelihood function for generalized linear models (Wedderburn, 1974) and was also used in Tan (2020b) and Tan (2020a) for fitting propensity score using high-dimensional data. In our study, the quasilikelihood function was chosen due to its alignment with the robust covariate balancing property exhibited by the corresponding quasi-score function (Ning et al., 2020). To establish the doubly robust confidence interval for ATE when analyzing high-dimensional data, the hyperparameter $\lambda_{\text{pooled}}^{\prime }$ in the (3) satisfied the following KKT condition

$${\left\|\frac{1}{Kn}\sum_{k=1}^{K}\sum_{i=1}^{n}\left\{\frac{T_{ki}}{\pi \left(X_{ki}^{T}{\overset{ˆ}{\theta }}_{\text{pooled}\;}\right)}-1\right\}X_{ki}\right\|}_{\infty }⩽\lambda_{\text{pooled}\;}^{\prime }.$$

This inequality implies that the maximum difference between the weighted average of $X_{ki}$ in the treatment group and the population average of $X_{ki}$ (e.g., $\frac{1}{Kn}\sum_{k=1}^{K}\sum_{i=1}^{n}X_{ki}$) is at most $\lambda_{\text{pooled}}^{\prime }$. Thus, the estimated propensity score $\pi (X_{ki}^{T}{\overset{ˆ}{\theta }}_{\text{pooled}})$ can approximately balance the covariates $X_{ki}$ (Tan, 2020a; Ning et al., 2020).

After ${\overset{ˆ}{\theta }}_{\text{pooled}}$ is obtained, we then estimate the parameter through the following global loss function of the outcome model:

(5) $${\overset{ˆ}{\beta }}_{\text{pooled}}=\underset{\theta \in R^{p}}{arg\;min}L_{N}(\beta ,{\overset{ˆ}{\theta }}_{\text{pooled}})+\lambda_{\text{pooled}}^{\prime \prime }{\left\|\beta \right\|}_{1},$$

where

(6) $$L_{N}(\beta ,\theta )=\frac{1}{Kn}\sum_{k=1}^{K}\sum_{i=1}^{N}\left\{\frac{T_{ki}}{exp\;\left(X_{ki}^{T}\theta \right)}{\left(Y_{ki}-X_{ki}^{T}\beta \right)}^{2}\right\}$$

is a weighted least square loss designed to achieve the desired doubly robust property. Finally, we obtain the AIPW estimate of $\tau_{1}^{*}=\frac{1}{K}\sum_{k=1}^{K}E\left[Y_{ki}(1)\right]$

$${\overset{ˆ}{\tau }}_{1,\text{pooled}}=\frac{1}{Kn}\sum_{k=1}^{K}\sum_{i=1}^{n}\left\{X_{ki}^{T}{\overset{ˆ}{\beta }}_{k}+\frac{T_{ki}}{\pi (X_{ki}^{T}{\overset{ˆ}{\theta }}_{k})}\left(Y_{ki}-X_{ki}^{T}{\overset{ˆ}{\beta }}_{k}\right)\right\}.$$

With the similar procedure, we estimate ${\overset{ˆ}{\tau }}_{0,\text{pooled}}$. Finally, we have:

$${\overset{ˆ}{\Delta }}_{1,\text{pooled}\;}={\overset{ˆ}{\tau }}_{1,\text{pooled}\;}-{\overset{ˆ}{\tau }}_{0,\text{pooled}}.$$

Regarding covariate shift, a notable aspect is that its presence does not affect the estimation of ATE in the pooled method. In other words, the existence of covariate shift does not introduce additional challenges when applying the introduced method for analyzing pooled data, as we assume the conditional distributions of Y are the same across all sites. Therefore, this method is treated as the gold standard method and will be compared with our method in simulation studies and data application.

### 2.2 Naive Method: Simple average method

Although ${\overset{ˆ}{\theta }}_{\text{pooled}}$ achieves the covariate balance property when analyzing the pooled data, in a distributed setting, where patient-level data cannot be shared across sites, the pooled estimator is not directly applicable. In such scenarios, an additional challenge arises: the decentralization of data. To tackle the complexities posed by high-dimensional and decentralized data analysis, a straightforward method is to aggregate the local estimates, which is a divide-and-conquer procedure. Specifically, each site fit the propensity score model and the outcome model:

(7) $${\overset{ˆ}{\theta }}_{k}=\underset{\theta \in R^{p}}{\arg min}Q_{j}(\theta )+\lambda^{\prime }{\left\|\theta \right\|}_{1},$$

(8) $${\overset{ˆ}{\beta }}_{k}=\underset{\theta \in R^{p}}{arg\;min}L_{k}\left(\beta ,{\overset{ˆ}{\theta }}_{k}\right)+\lambda^{\prime \prime }{\left\|\beta \right\|}_{1},$$

where $\lambda^{\prime }$ and $\lambda^{\prime \prime }$ are the regularization parameters, and

$$L_{k}\left(\beta ,\theta \right)=\frac{1}{n}\sum_{i=1}^{n}\left\{\frac{T_{ki}}{\exp\left(X_{ki}^{T}\theta \right)}{\left(Y_{ki}-X_{ki}^{T}\beta \right)}^{2}\right\}.$$

Within each site, we obtain the local AIPW estimate of $\tau_{1}^{*}$

$${\overset{ˆ}{\tau }}_{1,k}=\frac{1}{n}\sum_{i=1}^{n}\left\{X_{ki}^{T}{\overset{ˆ}{\beta }}_{k}+\frac{T_{ki}}{\pi (X_{ki}^{T}{\overset{ˆ}{\theta }}_{k})}\left(Y_{ki}-X_{ki}^{T}{\overset{ˆ}{\beta }}_{k}\right)\right\}.$$

Finally, we aggregate the local AIPW estimators

$${\overset{ˆ}{\tau }}_{1,\text{simple}\;\text{average}}=\frac{1}{K}\sum_{k=1}^{K}{\overset{ˆ}{\tau }}_{1,k}.$$

With the similar procedure, we estimate ${\overset{ˆ}{\tau }}_{0,\text{simple}\;\text{average}}$, and define ${\overset{ˆ}{\Delta }}_{1,\text{simple}\;\text{average}}={\overset{ˆ}{\tau }}_{1,\text{simple}\;\text{average}}-{\overset{ˆ}{\tau }}_{0,\text{simple}\;\text{average}}$. Although this method requires only a single round of communications to combine the local estimates, which have a convergence rate of $1/\sqrt{n}$, and the relatively small local sample sizes (i.e., n) could potentially lead to biased local estimates, which in turn could affect the overall accuracy of the ATE.

### 2.3 Proposed method: DisC^2^o-HD

In this paper, we present our method, DisC^2^o-HD, uniquely designed to collectively tackle all three challenges mentioned earlier – high-dimensional data, decentralized data, and covariate shift. The resolution of all these challenges is essential to bring federated causal learning into practical utility. In particular, motivated by the Taylor expansion of the likelihood function (Jordan et al., 2018), we proposed to construct a robust surrogate function of $Q_{N}(\theta )$ shown in the pooled method:

(9) $$\tilde{Q}\left(\theta ,\overset{‾}{\theta }\right)\;:=Q_{1}(\theta )+{\left(\nabla Q_{N}(\overset{‾}{\theta })-\nabla Q_{1}(\overset{‾}{\theta })\right)}^{T}\theta +\frac{1}{2}(\theta -\overset{‾}{\theta }{)}^{T}\left(\nabla^{2}Q_{N}(\overset{‾}{\theta })-\nabla^{2}Q_{1}(\overset{‾}{\theta })\right)(\theta -\overset{‾}{\theta }),$$

where $\overset{‾}{\theta }$ is an initial estimator of θ, for example, a meta-analysis estimator which required one more round of communication or a local estimator from the lead site, $Q_{1}(\theta )$, $\nabla Q_{1}(\overset{‾}{\theta })$, and $\nabla^{2}Q_{1}(\overset{‾}{\theta })$ are calculated within the 1-st site, also known as the lead site, assuming that we have full access to the patient-level data. For the rest of the sites from site 2 to K, they only need to communicate $\nabla Q_{k}(\overset{‾}{\theta })$, a p-dimensional vector, and $\nabla^{2}Q_{k}(\overset{‾}{\theta })$, a p×p matrix. We note that unlike the surrogate likelihood proposed by Jordan et al. (2018), $\tilde{Q}(\theta ,\overset{‾}{\theta })$ requires communicating the Hessian matrix $\nabla^{2}Q_{k}(\overset{‾}{\theta })$, which is essential to account for the covariate shift. A key procedure in Jordan et al. (2018) involves substituting the global Hessian matrix $\nabla^{2}Q_{N}(\theta )$, with a local Hessian matrix (e.g., Hessian matrix for Site 1), $\nabla^{2}Q_{1}(\theta )$. This substitution relies on the assumption:

$$\left\|\nabla^{2}Q_{1}(\theta )-\nabla^{2}Q_{N}(\theta )\right\|<\delta =o(1)$$

This indicates that the local datasets should be homogeneous from site to site. However, in settings characterized by heterogeneous data distribution, such as those involving covariate shifts — which are of particular interest to our study — this assumption may not hold. In other words, with the presence of covariate shift:

$$\left\|\nabla^{2}Q_{1}(\theta )-\nabla^{2}Q_{N}(\theta )\right\|=O(1)$$

Faced with such a scenario, if we do not account for the covariate shift, an additional constant term would be induced in the error bound of the surrogate estimators. More details are provided in the Supplementary Appendix 3.7. Therefore, we proposed using the average of all Hessian matrices from collaborating sites, rather than replacing the global Hessian matrix solely with the local one. This method ensures that:

$$\left\|\frac{1}{K}\sum_{k=1}^{K}\nabla^{2}Q_{k}(\theta )-\nabla^{2}Q_{N}(\theta )\right\|=0<\delta =o(1)$$

As a result, by collecting the Hessian matrices from the collaborating sites, the proposed method can address the issue of covariate shifts across different sites. This incorporation of Hessians shows that the presence of covariate shift does not affect the estimation of the ATE when using high-dimensional data. Then, we obtain the penalized surrogate propensity score estimator through

$$\tilde{\theta }=\underset{\theta \in R^{p}}{\arg min}\tilde{Q}(\theta ,\overset{‾}{\theta })+\lambda_{PS}{\left\|\theta \right\|}_{1},$$

where $\lambda_{PS}$ is a regularization parameter.

After we obtain the estimator, $\tilde{\theta }$, from the propensity score function, we need to further fit the outcome model in a distributed manner. Similarly, we construct the surrogate loss function:

(10) $$\begin{array}{l} \tilde{L}(\beta ,\overset{‾}{\beta },\tilde{\theta })=L_{1}(\beta )+{\left(\nabla L_{N}(\overset{‾}{\beta },\tilde{\theta })-\nabla L_{1}(\overset{‾}{\beta },\tilde{\theta })\right)}^{T}\beta \\ \;+\frac{1}{2}(\beta -\overset{‾}{\beta }{)}^{T}(\nabla^{2}L_{N}\left(\overset{‾}{\beta },\tilde{\theta }\right)-\nabla^{2}L_{1}\left(\overset{‾}{\beta },\tilde{\theta }\right))\left(\beta -\overset{‾}{\beta }\right), \end{array}$$

where $\overset{‾}{\beta }$ is an initial outcome model estimator. To construct $\tilde{L}(\beta ,\overset{‾}{\beta },\tilde{\theta })$ in (10), sites other than the lead site only need to contribute $\nabla L_{k}(\overset{‾}{\beta },\tilde{\theta })$ and $\nabla^{2}L_{k}(\overset{‾}{\beta },\tilde{\theta })$. Then, we compute the penalized surrogate outcome model estimator

$$\tilde{\beta }=\underset{\beta \in R^{p}}{\arg min}\tilde{L}(\beta ,\overset{‾}{\beta },\tilde{\theta })+\lambda_{OM}{\left\|\beta \right\|}_{1},$$

where $\lambda_{OM}$ is a regularization parameter.

In order to enhance the theoretical analysis, we utilize the sample splitting technique within our algorithms, a widely employed approach in semiparametric models and causal inference (Chernozhukov et al., 2018; Newey and Robins, 2018; van der Laan et al., 2011). Specifically, splitting data enables us to achieve independence between $\tilde{\theta },\;\tilde{\beta }$, and $X_{ki}$ in the final AIPW estimator. Consequently, the classical Bernstein Inequality for sub-exponential sums can be applied separately to bound each of them. Given the dilemma of sharing patient-level data, splitting patient-level data across sites into several folds requires additional rounds of communications. Furthermore, splitting patient-level data across sites results in a smaller sample size for obtaining the initial estimators of $\overset{‾}{\theta }$ and $\overset{‾}{\beta }$, which in turn can affect the convergence rate of the final AIPW estimator. Instead, we split the K sites into three sets $K_{1},\;K_{2}$, and $K_{3}$ with roughly equal size. This splitting strategy is shown to outperform the one by splitting patient-level data. For further discussion on the performance of splitting data versus not splitting it, as well as the comparison between splitting patient-level data and splitting sites, please refer to Section 4 in the supplementary material. In this section, we conduct additional numerical simulations to compare the performance between splitting and not splitting data, as well as between splitting patient-level data (split n) and splitting sites (split K).

To obtain the final average treatment effect (ATE), the following three steps are required.

- Step 1 involves conducting high-dimensional covariate balancing propensity score estimation using the aforementioned surrogate approach. The estimation is conducted in $K_{1},\;K_{2}$, and $K_{3}$, respectively.
- Step 2 entails fitting the outcome model in a distributed manner, employing a similar surrogate likelihood function approach. The estimation is conducted in $K_{1},\;K_{2}$, and $K_{3}$, respectively.
- Finally, in Step 3, we calculate the augmented inverse propensity weighted (AIPW) estimators from different splits and aggregate them to obtain the final ATE estimator.

For each step, we provide explicit algorithms, namely Algorithms 1, 2, and 3, respectively. In Algorithms 1 and 2, we show the algorithm in $K_{1}$ as an example. Same procedure is conducted for $K_{2}$ and $K_{3}$. The asymptotic variance of the final estimator ${\tilde{\tau }}_{1}$ in Algorithm 3, can be estimated through:

$$\hat{V}=\frac{1}{Kn}\sum_{k=1}^{K}\sum_{i=1}^{n}\frac{T_{ki}}{\pi {\left(X_{ki}^{T}\tilde{\theta }\right)}^{2}}{\left(Y_{ki}-X_{ki}^{T}\tilde{\beta }\right)}^{2}+\frac{1}{Kn}\sum_{k=1}^{K}\sum_{i=1}^{n}{\left(X_{ki}^{T}\tilde{\beta }-{\tilde{\tau }}_{1}\right)}^{2},$$

where $\tilde{\theta }=\left({\tilde{\theta }}_{K_{1}}+{\tilde{\theta }}_{K_{2}}+{\tilde{\theta }}_{K_{3}}\right)/3,\;\tilde{\beta }=\left({\tilde{\beta }}_{K_{1}}+{\tilde{\beta }}_{K_{2}}+{\tilde{\beta }}_{K_{3}}\right)/3$. The variance $\overset{ˆ}{V}$ can be computed in a distributed manner. In the following section, we present the theoretical justification of the proposed algorithm.

#### Remark 1

A key advantage of our proposed method is its ability to remain unaffected by the presence of covariate shift when estimating the ATE using high-dimensional data. In particular, we construct the surrogates of the propensity score model and outcome model with second order. These second-order surrogate models make use of the gradients and Hessians of the global objective functions, and approximate the higher-order derivatives of the global objective function by the counterparts of the objective function at the leading site, while the first-order surrogate likelihood method (Wang et al., 2017; Jordan et al., 2018) only keeps the gradient of the objective function and approximates all higher-order derivatives by the alternatives at the leading site.

As a comparison, the corresponding surrogate functions constructed from the idea in Jordan et al. (2018) are

(11) $${\tilde{Q}}^{\mathrm{original}}(\theta ,\overset{‾}{\theta })=Q_{1}(\theta )+{\left(\nabla Q_{N}(\overset{‾}{\theta })-\nabla Q_{1}(\overset{‾}{\theta })\right)}^{T}\theta ,$$

(12) $${\tilde{L}}^{\mathrm{original}}(\beta ,\overset{‾}{\beta },\tilde{\theta })=L_{1}(\beta )+{\left(\nabla L_{N}(\overset{‾}{\beta },\tilde{\theta })-\nabla L_{1}(\overset{‾}{\beta },\tilde{\theta })\right)}^{T}\beta .$$

*We refer the method that only necessitates the collection of first gradients across multiple sites to as the original surrogate method hereafter. We can similarly estimate ATE by plugging the penalized estimators using the original surrogate method. In our simulation studies and data application, we will compare our proposed method, denoted as DisC* ^2^ *o-HD-2, with this original surrogate method, denoted as DisC* ^2^ *o-HD-1.*

**Algorithm 1:** Distributed high-dimensional propensity score estimation on $K_{1}$

**Require:** $\left\{T_{ki},Y_{ki},X_{ki}\right\}$ for i=1,…,n, and $k\in K_{1}$.
At site k=1, calculate the initial propensity score estimator:
${\overset{‾}{\theta }}_{K_{1}}=\underset{\theta \in R^{p}}{\arg min}Q_{1}(\theta )+\lambda_{PS,\text{initial}}{\left\|\theta \right\|}_{1},$
where $Q_{1}(\theta )$ is defined in Equation (4) and $\lambda_{PS,\text{initial}}$ is the initial regularization parameter.
Broadcast ${\overset{‾}{\theta }}_{K_{1}}$ to all collaborating sites in $K_{1}$.
**for** site $k\in K_{1}$ **do**
Compute the first gradient $\nabla Q_{k}\left({\overset{‾}{\theta }}_{K_{1}}\right)$ and second gradient $\nabla^{2}Q_{k}\left({\overset{‾}{\theta }}_{K_{1}}\right)$. Broadcast these values to the leading site (i.e., k=1).
**end for**
Construct the surrogate loss $\tilde{Q}\left(\theta ,{\overset{‾}{\theta }}_{K_{1}}\right)$ in Equation (9), where
$\nabla Q_{N}\left({\overset{‾}{\theta }}_{K_{1}}\right)=\frac{1}{\left|K_{1}\right|}\sum_{k\in K_{1}}\nabla Q_{k}\left({\overset{‾}{\theta }}_{K_{1}}\right)$ and $\nabla^{2}Q_{N}\left({\overset{‾}{\theta }}_{K_{1}}\right)=\frac{1}{\left|K_{1}\right|}\sum_{k\in K_{1}}\nabla^{2}Q_{k}\left({\overset{‾}{\theta }}_{K_{1}}\right).$
Then, compute the penalized surrogate propensity score estimator
${\tilde{\theta }}_{K_{1}}=\underset{\theta \in RP}{arg\;min}\tilde{Q}\left(\theta ,{\overset{‾}{\theta }}_{K_{1}}\right)+\lambda_{PS}{\left\|\theta \right\|}_{1},$
where $\lambda_{PS}$ is a regularization parameter.

**Algorithm 2:** Distributed high-dimensional outcome model on $K_{1}$

**Require:** $\left\{T_{ki},Y_{ki},X_{ki}\right\}$ for i=1,…,n, and $k\in K_{1}$.
At site k=1, calculate the initial outcome model estimator:
${\overset{‾}{\beta }}_{K_{1}}=\underset{\theta \in R^{p}}{arg\;min}L_{1}\left(\beta ,{\tilde{\theta }}_{K_{2}}\right)+\lambda_{OM,\;\text{initial}\;}{\left\|\beta \right\|}_{1},$
Broadcast ${\overset{‾}{\beta }}_{K_{1}}$ to all collaborating sites in $K_{1}$.
**for** site $k\in K_{1}$ **do**
Compute the first gradient $\nabla L_{k}\left({\overset{‾}{\beta }}_{K_{1}},{\tilde{\theta }}_{K_{2}}\right)$ and second gradient $\nabla^{2}L_{k}\left({\overset{‾}{\beta }}_{K_{1}},{\tilde{\theta }}_{K_{2}}\right)$.
Broadcast these values to the leading site (i.e., k=1 )
**end for**
Construct the surrogate loss as defined in Equation (10). Then, compute the penalized surrogate outcome model estimator
${\tilde{\beta }}_{K_{1}}=\underset{\beta \in R^{p}}{arg\;min}\tilde{L}\left(\beta ,{\overset{‾}{\beta }}_{K_{1}},{\tilde{\theta }}_{K_{2}}\right)+\lambda_{OM}{\left\|\beta \right\|}_{1},$
where $\lambda_{OM}$ is a regularization parameter.

**Algorithm 3:** Calculation of AIPW estimators and final ATE

**Require:** $\left\{T_{ki},Y_{ki},X_{ki}\right\}$ for i=1,…,n, and k=1,…,K.
Calculate ${\tilde{\theta }}_{K_{1}}$ by Algorithm 1 on $K_{1}$ and broadcast ${\tilde{\theta }}_{K_{1}}$ to all sites in $K_{2}$ and $K_{3}$.
Calculate ${\tilde{\beta }}_{K_{2}}$ by Algorithm 2 on $K_{2}$ with ${\tilde{\theta }}_{K_{1}}$ and broadcast ${\tilde{\beta }}_{K_{2}}$ to all sites in $K_{3}$.
**for** site $k\in K_{3}$ **do**
Calculate the AIPW estimator of $\tau_{1}^{*}=E\left[Y_{ki}(1)\right]$
${\tilde{\tau }}_{1,k}=\frac{1}{n}\sum_{i=1}^{n}\left\{X_{ki}^{T}{\tilde{\beta }}_{K_{2}}+\frac{T_{ki}}{\pi \left(X_{ki}^{T}{\tilde{\theta }}_{K_{1}}\right)}\left(Y_{ki}-X_{ki}^{T}{\tilde{\beta }}_{K_{2}}\right)\right\}.$
**end for**
Aggregate the local AIPW estimators in $K_{3}$
${\tilde{\tau }}_{1,K_{3}}=\frac{1}{\left|K_{3}\right|}\sum_{k\in K_{3}}{\tilde{\tau }}_{1,k}.$
Calculate ${\tilde{\tau }}_{1,K_{1}}$ and ${\tilde{\tau }}_{1,K_{2}}$.
Calculate the final estimator of $\tau_{1}^{*}$ :
${\tilde{\tau }}_{1}=\left({\tilde{\tau }}_{1,K_{1}}+{\tilde{\tau }}_{1,K_{2}}+{\tilde{\tau }}_{1,K_{3}}\right)/3$

## 3 Theoretical Results

### 3.1 Assumptions

In this section, we present and discuss the assumptions under which our theoretical results are proved.

#### Assumption 1 (Unconfoundedness)

*The treatment assignment is unconfounded, i.e.*, $\left\{Y_{ki}(0),Y_{ki}(1)\right\}⫫T_{ki}|X_{ki}$.

#### Assumption 2 (Overlap)

*There exists a constant* $c_{0}>0$ *such that* $c_{0}\leq P\left(T_{ki}=1|X_{ki}\right)\leq 1-c_{0}$.

Assumption 1 requires that there is no unmeasured confounder. Assumption 2 implies that every sample has a positive probability to receive the treatment or belong to the control group. When Assumptions 1 and 2 are satisfied, the treatment assignment is considered as strongly ignorable (Rosenbaum and Rubin, 1983). The above two assumptions are standard in the causal inference literature.

#### Assumption 3 (Design)

The minimal and maximal eigenvalues of $E\left[X_{ki}X_{ki}^{T}\right]$ are contained in a bounded interval that does not contain zero.

Assumption 3 requires the design matrix is well conditioned. The same eigenvalue condition has been used to analyze high-dimensional lasso and causal inference problems (Van de Geer et al., 2014; Ning and Liu, 2017; Bradic et al., 2019). This assumption is utilized in Lemma 10, as detailed in the Supplementary Appendix Section 3.5 on the Restricted Strong Convexity (RSC) condition. It is a necessary condition for both the propensity score model and the outcome model in our proposed method. If site-specific covariates are present – for instance, a unique site indicator for each site – this assumption is violated, rendering the theoretical framework we have established for high-dimensional data inapplicable.

### 3.2 Restricted strong convexity(RSC) conditions

#### Assumption 4 (Model)

$X_{ki}$ *has a bounded sub-Gaussian norm. Moreover*, $\varepsilon_{ki}^{*}=Y_{ki}(1)-X_{ki}^{T}\beta^{*}$ *also has a bounded sub-Gaussian norm.*

Assumption 4 is a mild regularity condition on the tail of error term $\varepsilon_{ki}^{*}$ and design $X_{ki}$. This assumption controls the behavior of the error term and enables us to use various concentration inequalities in high-dimensional statistics.

#### Assumption 5 (Sparsity)

*Let* $s_{1}={\left\|\theta^{*}\right\|}_{0}$, *and* $s_{2}={\left\|\beta^{*}\right\|}_{0}$. *Assume that*

$$\frac{\sqrt{s_{2}\left(s_{1}\;\vee \;s_{2}\right)}\;log(p\;\vee \;Kn)}{\sqrt{Kn}}+\frac{s_{1}\sqrt{s_{1}s_{2}\;log(p\;\vee \;Kn)\;{log}^{4}(p\;\vee \;n)}}{n}=o(1)$$

*as* $s_{1},\;s_{2},\;p,\;K,\;n\to \infty$

Assumption 5 imposes conditions on how fast the model sparsity $s_{1}$ and $s_{2}$, the covariate dimension p and the number of sites K can grow with the local sample size n. When $s_{1}≍s_{2}≍s$, up to some logarithmic factors, the condition reduces to $\frac{s}{\sqrt{Kn}}+\frac{s^{2}}{n}=o(1)$. In addition, when K is fixed, it further reduces to $s/\sqrt{n}=o(1)$, which is identical to the existing sparsity conditions for high-dimensional treatment effect estimation (Tan, 2020a; Ning et al., 2020). Finally, we comment that the assumption may still hold even if $s_{1}$ is large but $s_{2}$ is small (more precisely, $s_{1}s_{2}$ is small), which is known as the sparsity double robustness property.

#### Assumption 6 (Variance)

*We assume that there exists some constant* $c_{1}>0$ *such that* $E\left(\varepsilon_{ki}^{*2}|X_{ki}\right)\geq c_{1},\;E{\left(X_{ki}^{T}\beta^{*}\right)}^{4}=O\left(s_{2}^{2}\right)$.

Assumption 6 is a mild condition on the noise and design. The first assumption guarantees the nondegeneracy of the asymptotic variance, while the second part is used in the Lyapunov condition in CLT.

### 3.3 Asymptotic distribution when both models are correctly specified

In this section, we present our main results with respect to the propensity score model, potential outcome model and the proposed estimator ${\tilde{\tau }}_{1}$, respectively. For simplicity, we use $C_{L}$ and M to denote some generic constants, whose values may differ from line to line.

For propensity score estimator ${\tilde{\theta }}_{K}$ obtained from Algorithm 1, we summarize its error bound in the following Proposition 1.

#### Proposition 1

*Under* assumptions 1–6, *with* $\lambda_{PS}≍\sqrt{\frac{log(p\vee Kn)}{Kn}}+\frac{s_{1}{log}^{2}(p\vee n)}{n}$, *we have:*

$$\begin{array}{rr} {\left\|{\tilde{\theta }}_{K}-\theta^{*}\right\|}_{2} & \leq C_{L}\left(\sqrt{\frac{s_{1}\;log(p\;\vee \;Kn)}{Kn}}+\frac{s_{1}^{3/2}{\;log}^{2}(p\;\vee \;n)}{n}\right),\\ \frac{3}{Kn}\sum_{(k,i)\in K}{\left\{X_{ki}^{T}({\tilde{\theta }}_{K}-\theta^{*})\right\}}^{2} & \leq C_{L}\left(\frac{s_{1}\;log(p\;\vee \;Kn)}{Kn}+\frac{s_{1}^{3}{\;log}^{4}(p\;\vee \;n)}{n^{2}}\right),\\ \frac{3}{Kn}\sum_{(k,i)\in K^{c}}{\left\{X_{ki}^{T}({\tilde{\theta }}_{K}-\theta^{*})\right\}}^{2} & \leq C_{L}\left(\frac{s_{1}\;log(p\;\vee \;Kn)}{Kn}+\frac{s_{1}^{3}{\;log}^{4}(p\;\vee \;n)}{n^{2}}\right). \end{array}$$

*holds with probability at least* $1-\frac{M}{{\left(p\vee n\right)}^{8}}-\frac{M}{n^{8}}$, *where* $K=K_{1},\;K_{2}$, *or* $K_{3}$ *and* $K^{c}$ *represents another set of servers.*

It can be seen that the error bound of ${\tilde{\theta }}_{K}$ consist of two terms. The first term is the classical lasso error bound when using the pooled data, signifying the optimal convergence rate achievable through this method. The second term can be represent by $s_{1}^{1/2}log(p\vee n){\left\|{\overset{‾}{\theta }}_{K}-\theta^{*}\right\|}_{2}^{2}$, which comes from the convergence rate of the initial local estimator ${\overset{‾}{\theta }}_{K}$. Consequently, it’s generally smaller than that of the local estimator ${\overset{‾}{\theta }}_{K}$, which is $\sqrt{\frac{s_{1}\;log(p\vee n)}{n}}$. While for the potential outcome estimator ${\tilde{\beta }}_{K}$, its error bound is provided as follows.

#### Proposition 2

*Under* assumptions 1–6, *with* $\lambda_{OM}≍\sqrt{\frac{log(p\vee Kn)}{Kn}}$, *we have:*

$$\begin{array}{ll} {\left\|{\tilde{\beta }}_{K}-\beta^{*}\right\|}_{2} & \leq C_{L}\left(\sqrt{\frac{s_{2}\log\left(p\;\vee \;Kn\right)}{Kn}}\right),\\ \frac{3}{Kn}\sum_{(i,j)\in K}{\left\{X_{ki}^{T}({\tilde{\beta }}_{K}-\beta^{*})\right\}}^{2} & \leq C_{L}\left(\frac{s_{2}\log\left(p\;\vee \;Kn\right)}{Kn}\right),\\ \frac{3}{Kn}\sum_{(i,j)\in K^{c}}{\left\{X_{ki}^{T}({\tilde{\beta }}_{K}-\beta^{*})\right\}}^{2} & \leq C_{L}\left(\frac{s_{2}\log\left(p\;\vee \;Kn\right)}{Kn}\right). \end{array}$$

*holds with probability at least* $1-\frac{M}{{\left(p\vee n\right)}^{8}}-\frac{M}{n^{8}}$, *where* $K=K_{1},\;K_{2}$, *or* $K_{3}$ *and* $K^{c}$ *represents another set of servers*.

We can see that the rate of ${\tilde{\beta }}_{K}$ is the same as that of the pooled estimator, which is obtained by directed applying the penalized loss function to the pooled data with the sample size Kn/3. This is due to that the objective function (6) is in a weighted least squares form. On one hand, the error in ${\tilde{\theta }}_{K}$ leads to a constant scaling of the weights, impacting only the constant scale but not the rate of convergence. On the other hand, a linear model has the lossless feature when using the surrogate likelihood method. This means that the distributed method can obtain exactly the same results as when the data are pooled together for analysis. Therefore, the convergence rate will not be affected by the initial local estimator ${\overset{‾}{\beta }}_{K}$. Specifically, the convergence rate of the Lasso estimator ${\tilde{\beta }}_{K}$ mainly depends on the infinity norm of the gradient, ${\left\|\nabla \tilde{L}(\beta^{*},{\overset{‾}{\beta }}_{K},{\tilde{\theta }}_{K^{c}})\right\|}_{\infty }$. Given that $\tilde{L}(\beta^{*},{\tilde{\theta }}_{K^{c}})$ represents a weighted least squares loss, by some calculation, it is easy to verify that $\nabla \tilde{L}(\beta^{*},{\overset{‾}{\beta }}_{K},{\tilde{\theta }}_{K^{c}})=\nabla L_{N}(\beta^{*},{\tilde{\theta }}_{K^{c}})$, thus the convergence rate of the initial estimator ${\overset{‾}{\beta }}_{K}$ does not affect the error bound of ${\left\|\nabla L_{N}(\beta^{*},{\tilde{\theta }}_{K^{c}})\right\|}_{\infty }$. Consequently, the error bound of ${\tilde{\beta }}_{K}$ shares the same order as that of the pooled estimator. Then, we present our main result on the bound of the proposed ATE estimator ${\tilde{\tau }}_{1}$ in the following two theorems.

#### Theorem 3

*Under* assumptions 1–6, *we have*

$$\left|{\tilde{\tau }}_{1}-{\overset{ˆ}{\tau }}_{1}^{*}\right|\leq C_{L}\left(\frac{\sqrt{s_{2}\left(s_{1}\;\vee \;s_{2}\right)}\;log(p\;\vee \;Kn)}{Kn}+\frac{\sqrt{s_{2}\left(s_{1}\;\vee \;s_{2}\right)\;log(p\;\vee \;Kn)\;{log}^{4}(p\;\vee \;n)}}{n\sqrt{Kn}}\right)$$

*with probability at least* $1-\frac{M}{{\left(p\;\vee \;n\right)}^{8}}-\frac{M}{n^{8}}$, *where*

$${\overset{ˆ}{\tau }}_{1}^{*}=\frac{1}{Kn}\sum_{k=1}^{K}\sum_{i=1}^{n}\left\{X_{ki}^{T}\beta^{*}+\frac{T_{ki}}{\pi \left(X_{ki}^{T}\theta^{*}\right)}\left(Y_{ki}-X_{ki}^{T}\beta^{*}\right)\right\}$$

is the asymptotic linear representation of the pooled AIPW estimator.

The first term in Theorem 3 represents the intrinsic error due to the estimation of nuisance parameters, which remains even if we are able to construct the pooled estimator by combining the data from multiple sites. Specifically, the order of the first term agrees with the findings of existing studies on the application of the hdCBPS estimator to pooled data with a sample size of Kn (Ning et al., 2020). The second term in Theorem 3 represents the cost incurred due to distributed learning. When K=o(n) holds, our distributed estimator is equivalent to the global estimator in terms of the error bound. Furthermore, in Theorem 4, we present our main result on the Berry-Esseen bound for the proposed estimator ${\tilde{\tau }}_{1}$.

#### Theorem 4

*Under* assumptions 1–6, *we have*

$$\begin{array}{l} \underset{x\in R}{sup}\left|Pr\left(\frac{\sqrt{Kn}\left({\tilde{\tau }}_{1}\;-\;\tau_{1}^{*}\right)}{\sqrt{\hat{V}}}\leq x\right)-\Phi (x)\right|\\ \leq \frac{M}{{\left(p\;\vee \;n\right)}^{8}}+\frac{M}{n^{8}}+C_{L}\left(\frac{\sqrt{s_{2}\left(s_{1}\;\vee \;s_{2}\right)}\;\log\left(p\;\vee \;Kn\right)}{\sqrt{Kn}}+\frac{s_{1}\sqrt{s_{1}s_{2}\;\log\left(p\;\vee \;Kn\right)\;\log^{4}\left(p\;\vee \;n\right)}}{n}\right), \end{array}$$

where $C_{L}$ is a sufficiently large constant, and M depends on $C_{L}$.

Theorem 4 implies the asymptotic normality of ${\tilde{\tau }}_{1}$, from which we can construct valid confidence intervals and hypothesis tests for $\tau_{1}^{*}$. Hahn (1998) proposed the semiparametric asymptotic variance bound for estimating $\tau_{1}^{*}$, that is

$$V^{*}:=E\left[\frac{1}{\pi_{ki}^{*}}E\left[\varepsilon_{ki}^{2}|X_{ki}\right]+{\left(X_{ki}^{T}\beta^{*}-\tau_{1}^{*}\right)}^{2}\right],$$

where $\pi_{ki}^{*}$ is the true value of the propensity score. We then show in Proposition 5 that the variance estimator defined in Theorem 4 consistently estimates $V^{*}$. Consequently, the proposed estimator ${\tilde{\tau }}_{1}$ achieves the semiparametric efficiency bound.

#### Proposition 5 (Consistency of variance estimator)

The variance estimator satisfies

$$\left|\hat{V}-V^{*}\right|\leq C_{L}\left(\sqrt{\frac{\left(s_{1}\;\vee \;s_{2}\right)\;log(p\;\vee \;Kn)}{Kn}}+\frac{s_{1}^{3/2}\;{log}^{2}(p\;\vee \;n)}{n}\right),$$

*with probability at least* $1-\frac{M}{{\left(p\;\vee \;n\right)}^{8}}-\frac{M}{n^{8}}$.

Throughout the various error bounds mentioned above, we find that $\frac{\sqrt{s_{2}\left(s_{1}\vee s_{2}\right)}log(p\vee Kn)}{\sqrt{Kn}}+\frac{s_{1}\sqrt{s_{1}s_{2}log(p\vee Kn){log}^{4}(p\vee n)}}{n}$ dominates all other terms above. Therefore, in Assumption 5, we typically assume that $\frac{\sqrt{s_{2}\left(s_{1}\vee s_{2}\right)}log(p\vee Kn)}{\sqrt{Kn}}+\frac{s_{1}\sqrt{s_{1}s_{2}log(p\vee Kn){log}^{4}(p\vee n)}}{n}=o(1)$, which implies that all the other terms are also o(1). Let $s=s_{1}\vee s_{2}$. Up to some logarithmic factors, this assumption can be reduced to $\frac{s}{\sqrt{Kn}}+\frac{s^{2}}{n}=o(1)$. This further simplifies to $s/\sqrt{n}=o(1)$ regardless of whether K is fixed or approaching infinity, which is identical to the existing sparsity conditions for high-dimensional treatment effect estimation Tan (2020a); Ning et al. (2020).

### 3.4 Asymptotic distribution when the models are misspecified

In this section, we first examine the robustness of the proposed estimator when the propensity score model is misspecified while the outcome model is correctly specified. In this setting, we assume that the true propensity score does not conform to the assumed parametric class, i.e., $P\left(T_{ki}=1|X_{ki}\right)\notin \left\{\pi \left(X_{ki}^{T}\theta \right):\theta \in R^{p}\right\}$. We define the estimand obtained through Algorithm 1 as follows:

$$\theta^{o}=\underset{\theta \in R^{d}}{arg\;min}E\left[Q_{k}(\theta )\right].$$

In the following, we demonstrate that the proposed estimator ${\tilde{\tau }}_{1}$ in Algorithm 3 is asymptotically equivalent to ${\overset{ˆ}{\tau }}_{1,\text{PS}}^{o}$ defined as follows

$${\overset{ˆ}{\tau }}_{1,PS}^{o}=\frac{1}{Kn}\sum_{k=1}^{K}\sum_{i=1}^{n}\left\{X_{ki}^{T}\beta^{*}+\frac{T_{ki}}{\pi \left(X_{ki}^{T}\theta^{o}\right)}\left(Y_{ki}-X_{ki}^{T}\beta^{*}\right)\right\},$$

where $\beta^{*}$ is the true value of β in the outcome model.

#### Proposition 6

*Under* Assumptions 1–6, *with* $\theta^{*}$ *replaced by* $\theta^{o}$, *the proposed estimator satisfies*

$$\left|{\tilde{\tau }}_{1}-{\overset{ˆ}{\tau }}_{1,PS}^{o}\right|\leq C_{L}\left(\frac{\sqrt{s_{2}\left(s_{1}\;\vee \;s_{2}\right)}\;log(p\;\vee \;Kn)}{Kn}+\frac{s_{1}\sqrt{s_{1}s_{2}\;log(p\;\vee \;Kn){\;log}^{4}(p\;\vee \;n)}}{n\sqrt{Kn}}\right)$$

*with probability at least* $1-\frac{M}{n^{8}}$, *where* $C_{L}$ *is a sufficiently large constant and* M *is another constant depending on* $C_{L}$.

Since ${\overset{ˆ}{\tau }}_{1,PS}^{o}$ is asymptotically normal with mean $\tau_{1}^{*}$, we can establish the asymptotic normality of the proposed estimator ${\tilde{\tau }}_{1}$. Consequently, the resulting confidence intervals remain valid even under the misspecified propensity score model. This provides justification for the robustness of the confidence intervals (Tan, 2020a; Ning et al., 2020).

We then can examine the robustness of the proposed estimator when the propensity score model is correctly specified while the outcome model is misspecified. In this scenario, we assume that the true potential outcome is nonlinear, meaning that $\left[Y_{ki}(1)|X_{ki}\right]\notin \left\{X_{ki}\beta :\beta \in R^{p}\right\}$. Next, we define the estimand obtained through Algorithm 2 as follows:

$$\beta^{o}=\underset{\beta \in R^{d}}{arg\;min}E[L_{k}(\beta ,\theta^{*})],$$

where $\theta^{*}$ is the true value in the propensity score model. Define ${\overset{ˆ}{\tau }}_{1,OM}^{o}$ as

$${\overset{ˆ}{\tau }}_{1,OM}^{o}=\frac{1}{Kn}\sum_{k=1}^{K}\sum_{i=1}^{n}\left\{X_{ki}^{T}\beta^{o}+\frac{T_{ki}}{\pi \left(X_{ki}^{T}\theta^{*}\right)}\left(Y_{ki}-X_{ki}^{T}\beta^{o}\right)\right\}.$$

#### Proposition 7

*Under* Assumptions 1–6, *with* $\beta^{*}$ *replaced by* $\beta^{o}$, *the proposed estimator satisfies*

$$\left|{\tilde{\tau }}_{1}-{\overset{ˆ}{\tau }}_{1,OM}^{o}\right|\leq C_{L}\left(\frac{\sqrt{s_{2}\left(s_{1}\;\vee \;s_{2}\right)}\;log(p\;\vee \;Kn)}{Kn}+\frac{s_{1}\sqrt{s_{1}s_{2}\;log(p\;\vee \;Kn){\;log}^{4}(p\;\vee \;n)}}{n\sqrt{Kn}}\right)$$

*with probability at least* $1-\frac{M}{n^{8}}$, *where* $C_{L}$ *is a sufficiently large constant and* M *is another constant depending on* $C_{L}$.

Similarly, we demonstrate that the proposed estimator ${\tilde{\tau }}_{1}$ in Algorithm 3 is asymptotically equivalent to ${\overset{ˆ}{\tau }}_{1,OM}^{o}$. This equivalence implies the robustness of confidence intervals even in the presence of misspecified outcome models.

## 4 Simulation studies

In this section, we examine the performance of the proposed DisC^2^o-HD-2 estimator by comparing them with the pooled estimator, the local average method, and the DisC^2^o-HD-1 estimator. Without loss of generality, for k=1,…,K and i=1,…,n, the treatment $T_{ki}$ are generated from a logistic regression with $\pi_{ki}=expit\left(-0.5+0.5X_{ki1}+0.3X_{ki2}-0.3X_{ki3}+\right.0.3X_{ki4}-0.3X_{ki5})$, the potential outcomes satisfy $Y_{ki}(1)=2+0.3X_{ki1}+0.2X_{ki2}-0.2X_{ki3}+$ $0.2X_{ki4}-0.2X_{ki5}+\epsilon_{ki1}$ and $Y_{ki}(0)=1+0.3X_{ki1}+0.2X_{ki2}-0.2X_{ki3}+0.2X_{ki4}-0.2X_{ki5}+\epsilon_{ki0}$, where $\epsilon_{ki1}$ and $\epsilon_{ki0}$ are i.i.d from N(0,1), while the p-dimensional covariates are generated from $X_{ki}\sim N\left(0,\Sigma_{k}\right)$. We consider the following seven scenarios.

- **Homogeneous covariates with** p<n: We consider the dimension with p=100 and the sample size in each site is fixed at n=200, while the covariance matrix $\Sigma_{k}$ is set to be $\Sigma_{k;st}=0.5^{\left|s-t\right|}$ for k=1,…,K. In this case, the simple size is larger than the dimension, and the distribution of covariates is homogeneous across sites.
- **Heterogeneous covariates (i.e., covariate shift) with** p<n: We consider the dimension with p=100 and the sample size in each site is fixed at n=200, while the covariance matrix $\Sigma_{k}$ is set to be $\Sigma_{k;st}=\rho_{k}^{\left|s-t\right|}$, where $\rho_{k}\sim Uniform(0.2,\;0.8)$ for k=1,…,K. In this case, the simple size is larger than the dimension, and there is a shift in the distribution of covariates across sites.
- **Homogeneous covariates with** p>n: We consider the dimension with p=500 and the sample size in each site is fixed at n=200, while the covariance matrix $\Sigma_{k}$ is set to be $\Sigma_{k;st}=0.5^{\left|s-t\right|}$ for k=1,…,K. In this case, the simple size is smaller than the dimension, and the distribution of covariates is homogeneous across sites.
- **Heterogeneous covariates with** p>n: We consider the dimension with p=500 and the sample size in each site is fixed at n=200, while the covariance matrix $\Sigma_{k}$ is set to be $\Sigma_{k;st}=\rho_{k}^{\left|s-t\right|}$, where $\rho_{k}\sim Uniform(0.2,\;0.8)$ for k=1,…,K. In this case, the simple size is smaller than the dimension, and there is a shift in the distribution of covariates across sites.
- **Misspecified propensity score model with** p>n: We further consider the transformed covariates $X_{ki,mis}=(X_{ki,1},X_{ki,2},X_{ki,3}^{3},exp\left(X_{ki,4}\right),X_{ki,5}{\left(1+exp\left(X_{ki,6}\right)\right)}^{-2},\left.X_{ki,7},\ldots ,X_{ki,p}\right)$. In this case, the treatment $T_{ki}$ is generated from the logistic regression in (IV) with $X_{ki}$ replaced with the transformed covariates $X_{ki,mis}$, while the potential outcomes are generated in the same way as in (IV).
- **Misspecified outcome model with** p>n: We consider the same transformed covariates as in (V). In this case, the potential outcomes $y_{ki}$ is generated from the linear regression in (IV) with $X_{ki}$ replaced with the transformed covariates $X_{ki,mis}$, while the treatments are generated in the same way as in (IV).
- **Misspecified propensity score and outcome models with** p>n: We consider the same transformed covariates as in (V). In this case, both the treatment and potential outcomes are generated from the models in (IV)with $X_{ki}$ replaced with the transformed covariates $X_{\text{ki},\text{mis}}$.

In each scenario, we repeat the simulation 100 times and vary the number of sites K in {10, 20, 30, 40, 50, 60} to mimic research networks with moderate to large size, respectively. The regularization parameters in both algorithms are selected via cross-validation.

**Figure 2:**
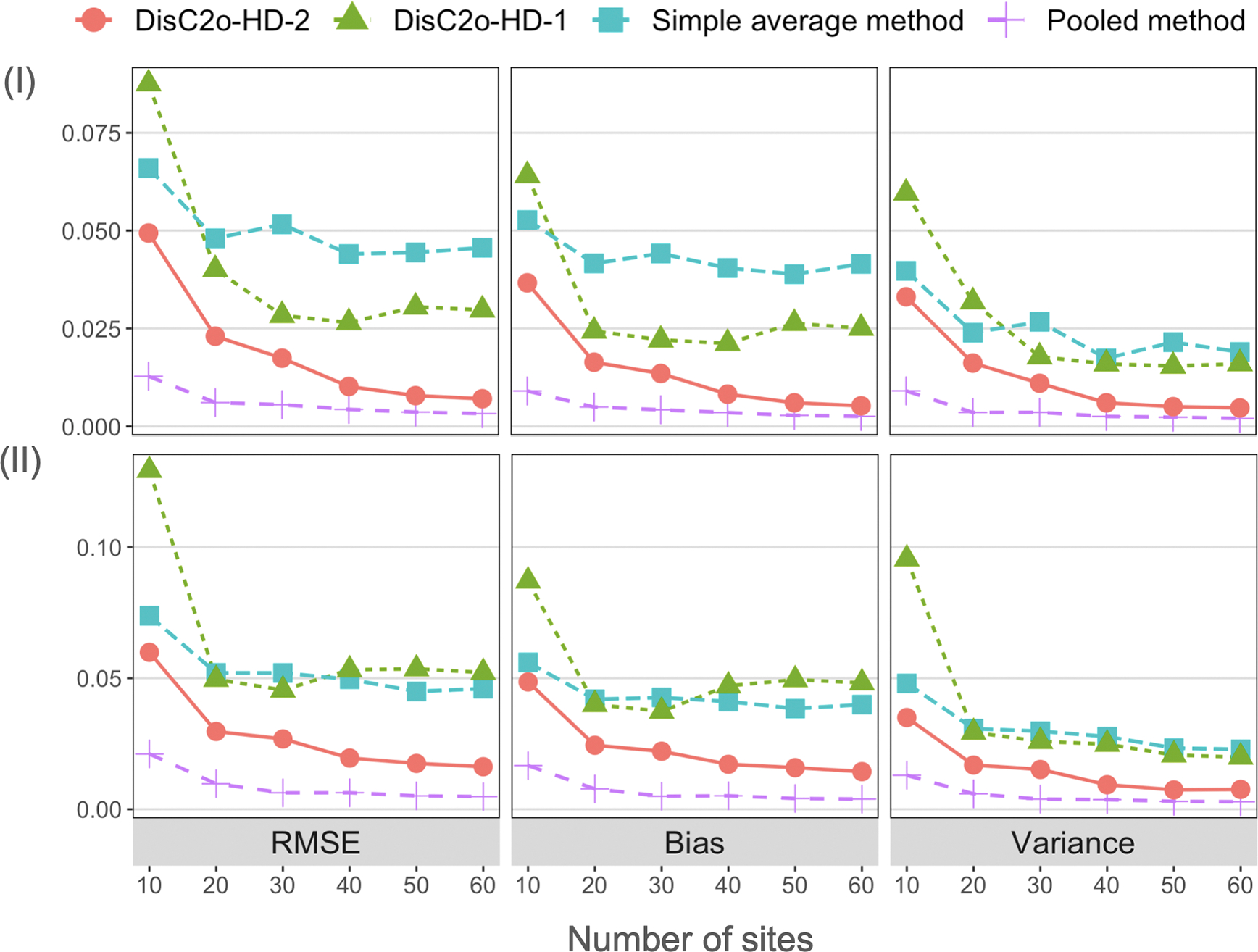
Simulation results for the lower-dimensional settings (i.e., *p* < *n*). Upper panel: comparison results of different methods under scenario (I) – homogeneous covariates with *p* < *n*; lower panel: comparison results of different methods under scenario (II) – heterogeneous covariates with *p* < *n*

**Figure 3:**
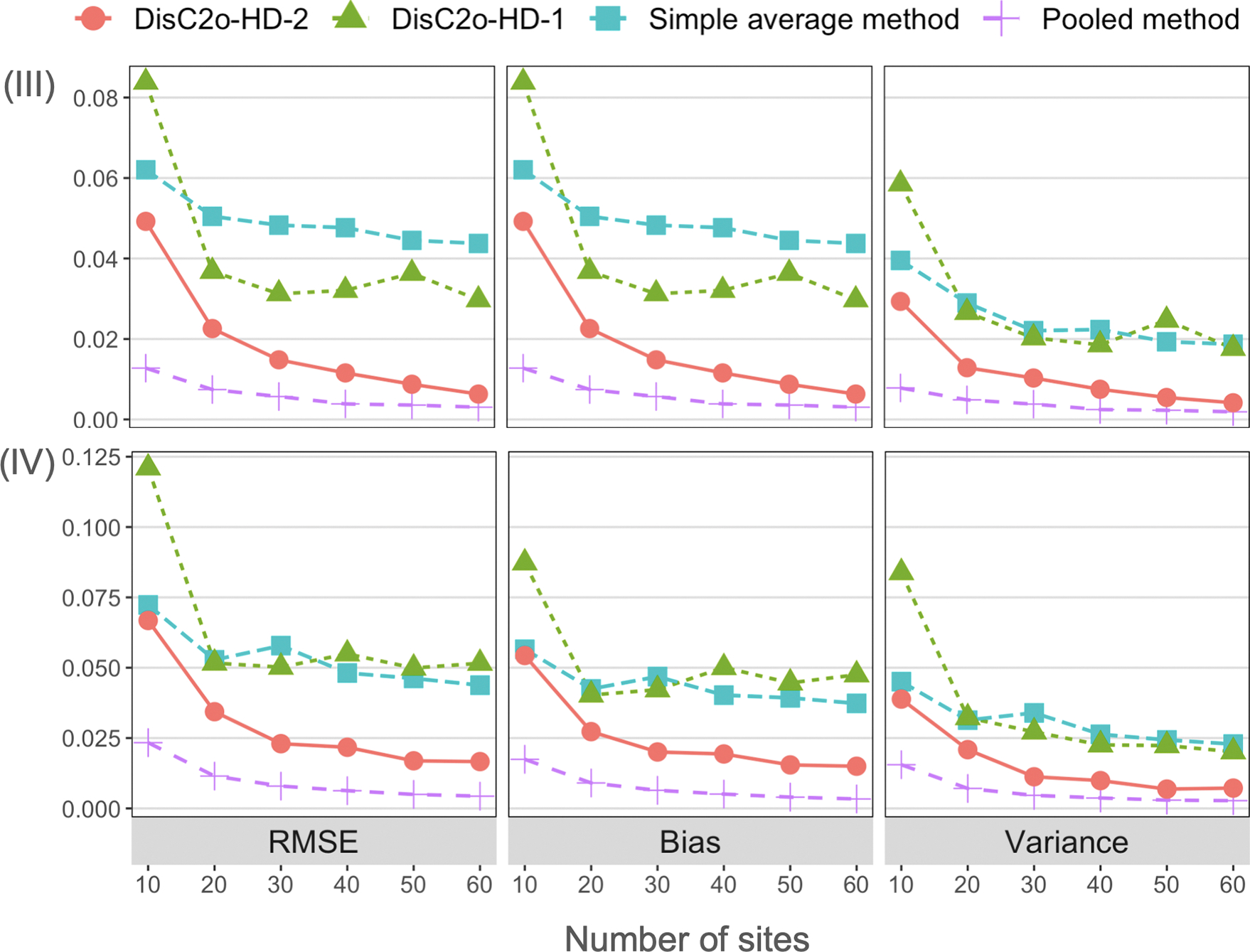
Simulation results for the high-dimensional settings (i.e., *p* > *n*). Upper panel: comparison results of different methods under scenario (III) – homogeneous covariates with *p* > *n*; lower panel: comparison results of different methods under scenario (IV) – heterogeneous covariates with *p* > *n*

**Figure 4:**
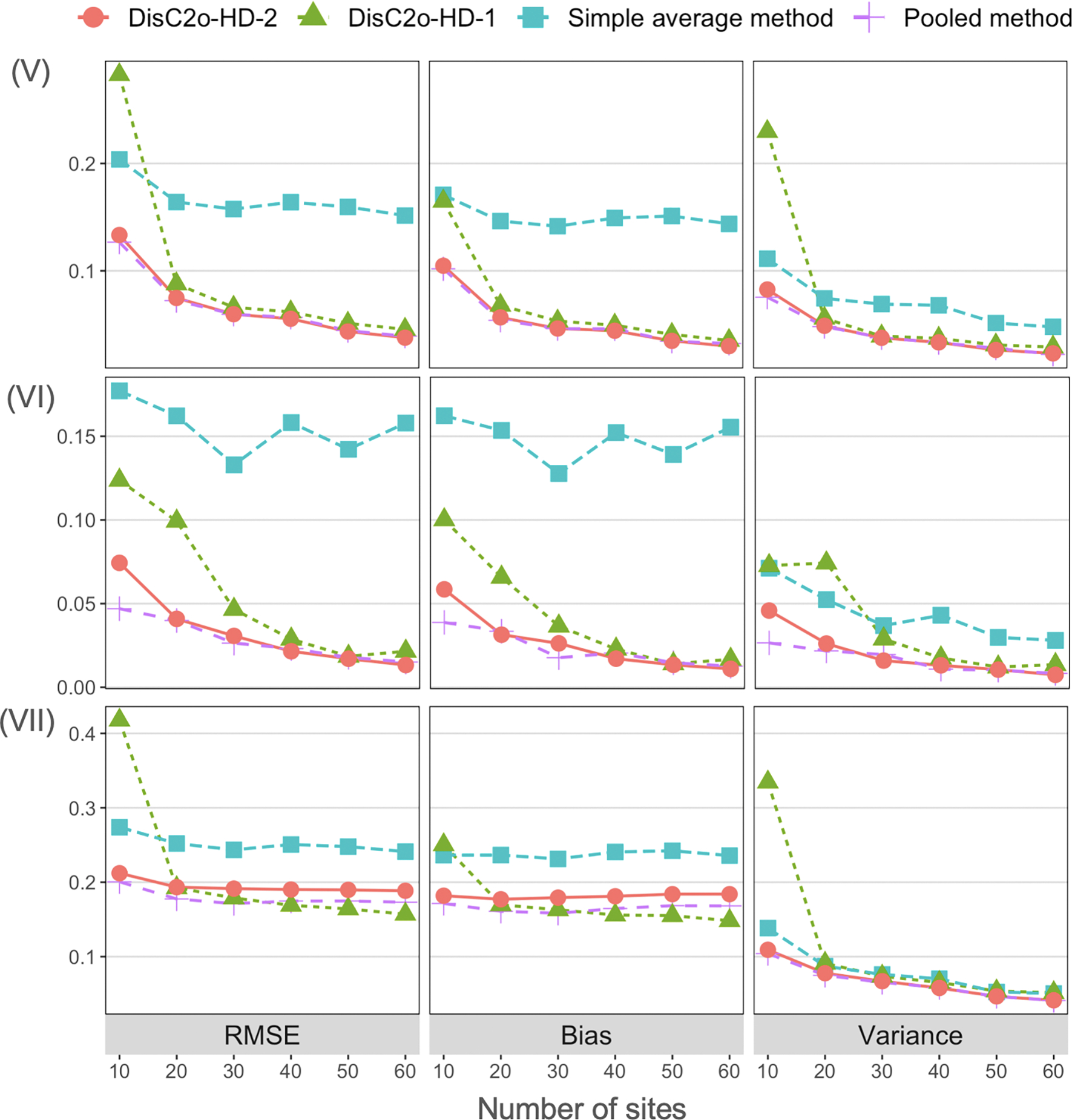
Simulation results for the high-dimensional settings with model misspecification. Upper panel: comparison results of different methods under scenario (V) – misspecified propensity score model with *p* > *n*; middle panel: comparison results of different methods under scenario (VI) – misspecified outcome model with *p* > *n*; lower panel: comparison results of different methods under scenario (VI) – misspecified propensity score model and outcome model with *p* > *n*

We compare the proposed DisC^2^o-HD-2 approach and other approaches in terms of the root-mean-squared error (RMSE), absolute value of bias, variance under all scenarios. The comparison results of correctly specified cases are present in Figure 2 and 3. The four figures show that the proposed DisC^2^o-HD-2 approach tends to have smaller bias and variance and hence have significantly smaller RMSE than that of other approaches except the pooled estimator in all scenarios. In addition, as the number of sites increases, the RMSE, bias, and variance of the proposed method decrease accordingly. However, the local average method and DisC^2^o-HD-1 are less robust as the number of sites increases. In addition, the proposed DisC^2^o-HD-2 approach is robust to the model misspecification and usually outperforms other methods as shown in Figure 4.

In summary, under the distributed setting, the proposed DisC^2^o-HD-2 approach demonstrates superior performance and closely approximates the pooled results as the number of sites (*K*) increases. The proposed approach also exhibits more robust performance under model misspecifications.

## 5 Data application

A number of studies have been conducted to investigate the long-term consequences of SARS-CoV-2, the virus responsible for COVID-19. Post-acute sequelae of SARS-CoV-2, hereafter referred to as PASC, can manifest as various health issues affecting multiple organ systems, appearing four weeks or more after infection. The World Health Organization has defined post-COVID-19 conditions as those occurring three months after the initial infection, lasting a minimum of two months, and lacking an alternative diagnosis. However, there is limited information regarding the impact of the vaccine on PASC in diverse pediatric populations, particularly children in the United States.

In this section, we assess the proposed methods by utilizing electronic health records (EHR) data obtained from the Children’s Hospital of Philadelphia. Our objective is to investigate the impact of the vaccine on Post-Acute Sequelae of SARS-CoV-2 infection (PASC) in children during the Omicron period. The data used for analysis were extracted from the PEDSnet COVID-19 Database Week 129 and encompassed EHR data with service dates up until November 30, 2022. The database encompasses data from over 5,000 clinical care providers in the Greater Philadelphia area, originating from numerous outpatient centers in the western suburbs, Central Pennsylvania, and New Jersey or New York City. Within the EHR data, we had access to a comprehensive assortment of routinely collected clinical information, including patient demographics, medication records, coded procedures and diagnoses, medical history, allergies, laboratory results, microbiology, blood bank information, pathology reports, vital signs, surgical records, and anesthesia details, among other data elements.

The eligibility criteria for participants in this study included being between the ages of 5 and 11 at the beginning of the study period, with no previous COVID-19 vaccination or documented SARS-CoV-2 infection. Additionally, participants were required to have a prior encounter (including telephone or telehealth encounters) within 18 months before entering the cohort to ensure an ongoing interaction with the healthcare system. The intervention under investigation was vaccination, specifically comparing those who received any type of COVID-19 vaccine with those who did not receive any. The outcome of interest was the count of PASC features observed within 28 to 179 days following the initial SARS-CoV-2 test date.

From patients’ medical records, we identified a set of confounders and extracted relevant information. These confounders encompassed demographic variables such as age and gender, race, obesity status, Pediatric Medical Complexity Algorithm (PMCA) score (Simon et al., 2018), the number of visits to the emergency department in the 18 months leading up to 7 days before cohort entry, the number of inpatient visits during the same time frame, the number of outpatient visits within the specified period, the count of unique medications prescribed within the 18 months prior to 7 days before cohort entry, and the presence of diagnoses related to 205 chronic condition clusters within the same timeframe. In total, we included 248 confounders in our analysis.

**Figure 5:**
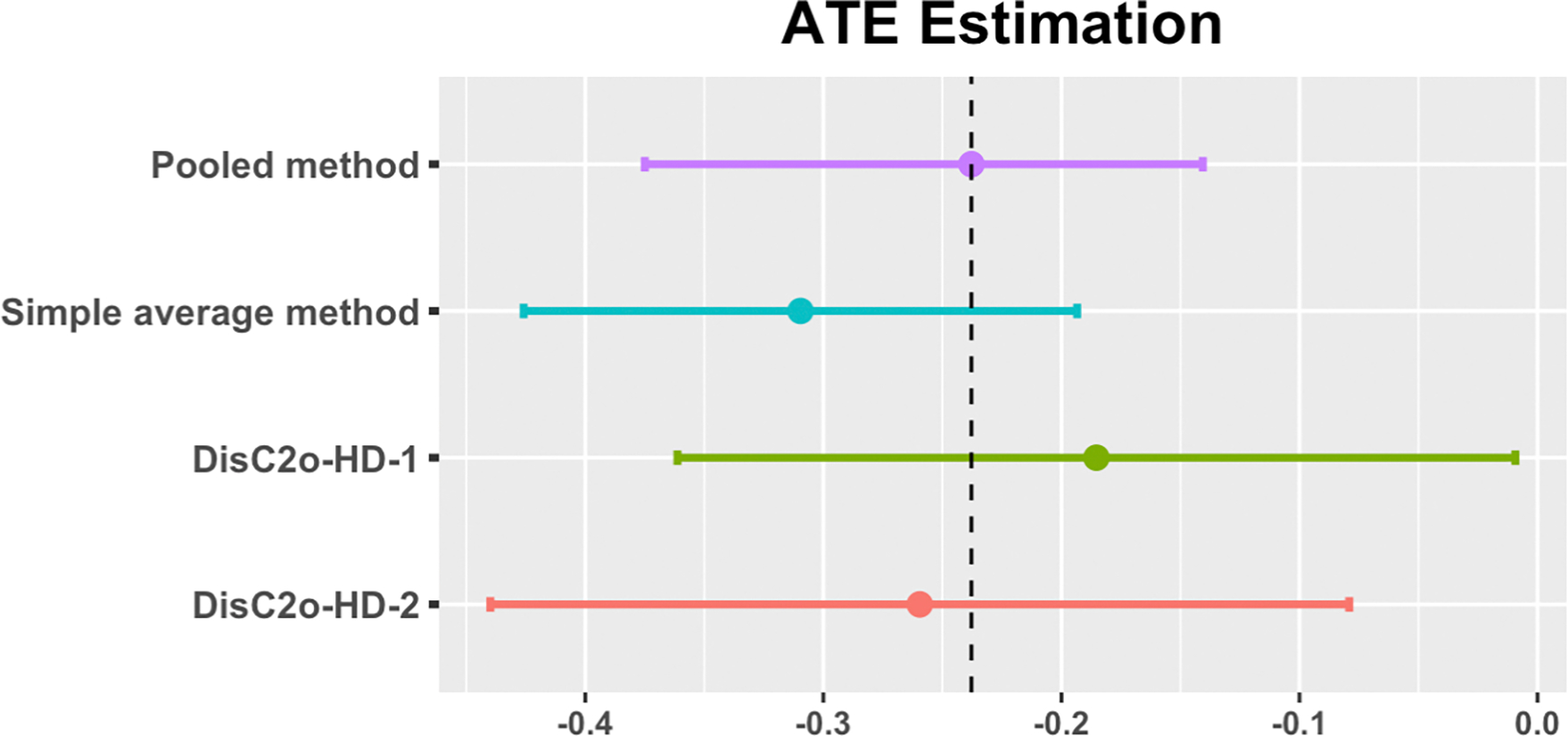
Data analysis results on the investigation on the impact of the vaccine on Post-Acute Sequelae of SARS-CoV-2 infection (PASC) in children during the Omicron period.

After implementing data quality control measures, our final dataset consisted of 1,158 individuals. These individuals were collected from 6 clinical sites, with each site contributing approximately 193 patients to the analysis. We have complete access to all 1,158 individuals, enabling us to apply both pooled analysis and the proposed method to the dataset. Figure 5 illustrates the results of the data analysis, comparing the pooled method, simple average method, DisC^2^o-HD-1, and the proposed method. Among these methods, the proposed method (highlighted in red) yields the ATE estimation of −0.26 (95% CI: [−0.44, −0.08]) closest to that of the pooled method estimation of −0.24 (95% CI: [−0.38, −0.14]) (highlighted in purple), which is considered as the gold standard. It is worth noting that the proposed method exhibits a loss in efficiency, resulting in a wider confidence interval for the estimate. The results of this study by examining the impact of COVID-19 vaccination on children aged 5 to 11 showed consistent findings with previous studies conducted on adults (Wynberg et al., 2022). However, further investigations are warranted to better understand the effects of vaccination on children in this age group.

## 6 Conclusion

Overall, this paper presents a novel approach to address the challenges of high-dimensional healthcare data analysis, offering a distributed learning algorithm that effectively accounts for covariate shift and enables accurate estimation of the average treatment effect. The proposed method shows promise for improving healthcare research and decision-making by leveraging large-scale data from multiple clinical sites. The implementation of our proposed method requires a uniform set of covariates across all participating sites to ensure the validity of statistical analyses. However, we recognize that this requirement could limit its applicability in scenarios with structural missingness. Therefore, it is crucial for future extensions to address the presence of structural missingness in multi-site studies when analyzing high-dimensional data. Additionally, we plan to extend this method for other types of outcomes and address the potential issue of small sample sizes of the collaborating clinical sites. It is also important to note that although we assume the coefficients are homogeneous across sites in our models, these coefficients could indeed vary across different sites, particularly when analyzing complex, real-world, multi-site data. Some efforts have been made in the field of distributed learning. For example, Duan et al. (2022) proposed using the density ratio tilting method to accommodate differences in coefficients. We look forward to extending our current framework to more comprehensively understand and account for the potential variability of coefficients in the model. In addition to calculating the ATE at the population level, it is essential to assess the site-specific ATE. This analysis can yield significant insights into the factors driving heterogeneity across clinical sites.

## Supplementary Material

Supplement
