# Supplementary material for: DisC2o-HD: Distributed causal inference with covariates shift for analyzing real-world high-dimensional data
Source: J Mach Learn Res. Author manuscript; Available in PMC 2025 Jul 17. (PMC12269483)
Supplement: Supplement [file NIHMS2087339-supplement-Supplement.pdf]

DisC^{2}o-HD: Distributed causal inference with covariates shift for analyzing real-world high-dimensional data: Supplementary Materials

1 Assumptions

Recall that the loss functions for DisC^{2}o-HD estimator is defined as:

$$\tilde{Q}(\theta,\bar{\theta}) = Q_1(\theta) + \left(\nabla Q_N(\bar{\theta}) - \nabla Q_1(\bar{\theta})\right)^T \theta + \frac{1}{2}(\theta-\bar{\theta})^T \left(\nabla^2 Q_N(\bar{\theta}) - \nabla^2 Q_1(\bar{\theta})\right)(\theta-\bar{\theta}),$$

$$\tilde{L}(\beta,\bar{\beta},\tilde{\theta}_{K^c}) = L_1(\beta) + \left(\nabla L_N(\bar{\beta},\tilde{\theta}_{K^c}) - \nabla L_1(\bar{\beta},\tilde{\theta}_{K^c})\right)^T \theta + \frac{1}{2}(\beta-\bar{\beta})^T \left(\nabla^2 L_N(\bar{\beta},\tilde{\theta}_{K^c}) - \nabla^2 L_1(\bar{\beta},\tilde{\theta}_{K^c})\right)(\theta-\bar{\theta}),$$

respectively. Where $\bar{\theta}$ and $\bar{\beta}$ are proper initial estimator.

In this section, we present and discuss the assumptions under which our theoretical results are proved.

Assumption 1 (Unconfoundedness)**.** *The treatment assignment is unconfounded, i.e.,* $\{Y_{ki}(0), Y_{ki}(1)\} \perp\!\!\!\perp T_{ki} \mid X_{ki}$.

Assumption 2 (Overlap)**.** *There exists a constant* $c_0 > 0$ *such that* $c_0 \leqslant \mathbb{P}(T_{ki} = 1|X_{ki}) \leqslant 1 - c_0$.

Assumption 1 requires that there is no unmeasured confounder. Assumption 2 implies that every sample has a positive probability to receive the treatment or belong to the control group. When Assumptions 1 and 2 are satisfied, the treatment assignment is considered as strongly ignorable (Rosenbaum and Rubin, 1983). The above two assumptions are standard in the causal inference literature.

Assumption 3 (Design)**.** *The minimal and maximal eigenvalues of* $\mathbb{E}[X_{ki}X_{ki}^T]$ *are contained in a bounded interval that does not contain zero.*

Assumption 3 requires the design matrix is well conditioned. The same eigenvalue condition has been used to analyze high-dimensional lasso and causal inference problems (Van de Geer et al., 2014; Ning and Liu, 2017; Bradic et al., 2019).

Assumption 4 (Model)**.** X_{ki} *has mean* 0 *and a bounded sub-Gaussian norm. Moreover,* $\varepsilon_{ki}^* = Y_{ki}(1) - X_{ki}^T\beta^*$ *also has a bounded sub-Gaussian norm.*

Assumption 4 is a mild regularity condition on the tail of error term ε_{ki}^* and design X_{ki}. This assumption controls the behavior of the error term and enables us to use various concentration inequalities in high-dimensional statistics. Since the mean shift of X_{ki} does not influence the procedure of the proof, without loss of generality, we further assume that X_{ki} has a mean of 0 for the convenience of the proof, even though it may vary across sites.

Assumption 5 (Sparsity)**.** *Let* $s_1 = \|\theta^*\|_0$, *and* $s_2 = \|\beta^*\|_0$. *Assume that*

$$\frac{\sqrt{s_2(s_1 \vee s_2)}\log(p \vee Kn)}{\sqrt{Kn}} + \frac{s_1\sqrt{s_1 s_2 \log(p \vee Kn)\log^4(p \vee n)}}{n} = o(1)$$

as $s_1, s_2, p, m, n \to \infty$.

Assumption 5 imposes conditions on how fast the model sparsity s_1 and s_2, the covariate dimension p and the number of sites K can grow with the local sample size n. When $s_1 \asymp s_2 \asymp s$, upto some logarithmic factors, the condition reduces to $\frac{s}{\sqrt{Kn}} + \frac{s^2}{n} = o(1)$. In addition, when K is fixed, it further reduces to $s/\sqrt{n} = o(1)$, which is identical to the existing sparsity conditions for high-dimensional treatment effect estimation (Tan, 2020; Ning et al., 2020). Finally, we comment that the assumption may still hold even if s_1 is large but s_2 is small (more precisely, $s_1 s_2$ is small). This is known as the sparsity double robustness property, recently proposed by Bradic et al. (2019).

Assumption 6 (Variance)**.** *We assume that there exists some constant* $c_1 > 0$ *such that* $\mathbb{E}(\varepsilon_{ki}^{*2}|X_{ki}) \geqslant c_1$, $\mathbb{E}\left(X_{ki}^T\beta^*\right)^4 = O(s_2^2)$.

Assumption 6 is a mild condition on the noise and design. The first assumption guarantees the nondegeneracy of the asymptotic variance, while the second part is used in the Lyapunov condition in CLT.

2 Notation

Throughout this paper, we use the following notation. For $v = (v_1, \ldots, v_p) \in \mathbb{R}^p$ and $1 \leqslant q \leqslant \infty$, we define $\|v\|_q = \left(\sum_{i=1}^d |v_i|^q\right)^{1/q}$, $\|v\|_0 = |\text{supp}(v)|$, in which $\text{supp}(v) = \{j : v_K \neq 0\}$. The Orlicz norm associated with a Young's modulus ψ of X is defined by $\|X\|_\psi = \inf\{C > 0 : \mathbb{E}[\psi(|X|/C)] \leqslant 1\}$. If a matrix A is symmetric, then $\lambda_{\text{min}}(A)$ and $\lambda_{\text{max}}(A)$ represent the minimal and maximal eigenvalues of A. For two positive sequences a_n and b_n, we write $a_n \asymp b_n$ if there exist $C, C' > 0$ such that $C \leqslant a_n/b_n \leqslant C'$ holds. We denote $\psi_1 = e^{x^2} - 1$ and if a random variable X is sub-Gaussian, then $\|X\|_{\psi_1} < \infty$. Denote $a \vee b = \max(a, b)$.

3 Detailed proof

Throughout the proof below, we denote J to be a set of the servers, while K^c to be another set of servers. As an example, when J is the K_1, then K^c can be either K_2 or K_3.

3.1 Main result

Proposition 1. *Under assumption 1-6, with* $\lambda_{ps} \asymp \sqrt{\frac{\log(p\vee Kn)}{Kn}} + \frac{s_1\log^2(p\vee n)}{n}$, *for DisC*^{2}*o-HD estimator* $\tilde{\theta}_K$, *we have:*

$$\left\|\tilde{\theta}_K - \theta^*\right\|_2 \leqslant C_L\left(\sqrt{\frac{s_1\log(p\vee Kn)}{Kn}} + \frac{s_1^{3/2}\log^2(p\vee n)}{n}\right),$$

$$\frac{3}{Kn}\sum_{(k,i)\in K}\left\{X_{ki}^T(\tilde{\theta}_K - \theta^*)\right\}^2 \leqslant C_L\left(\frac{s_1\log(p\vee Kn)}{Kn} + \frac{s_1^3\log^4(p\vee n)}{n^2}\right),$$

$$\frac{3}{Kn}\sum_{(k,i)\in K^c}\left\{X_{ki}^T(\tilde{\theta}_K - \theta^*)\right\}^2 \leqslant C_L\left(\frac{s_1\log(p\vee Kn)}{Kn} + \frac{s_1^3\log^4(p\vee n)}{n^2}\right).$$

holds with probability at least $1 - \frac{M}{(p\vee n)^8} - \frac{M}{n^8}$, *where* $K = K_1$, K_2, *or* K_3 *and* K^c *represents another set of servers.*

Proof. **First Claim:**

Consider the events defined as:

$$\mathcal{E}_1 = \left\{\|\bar{\theta}_K - \theta^*\|_2 \leqslant C_1\sqrt{\frac{s_1\log(p\vee n)}{n}}\right\},$$

$$\mathcal{E}_2 = \left\{\|\nabla Q_N(\theta^*)\|_\infty \leqslant C_1\sqrt{\frac{\log(p\vee Kn)}{Kn}}\right\},$$

$$\mathcal{E}_3 = \left\{\max_K \|X_{i1}\|_\infty \leqslant C\log(p\vee n)\right\},$$

$$\mathcal{E}_4 = \left\{\frac{1}{n}\sum_{(i,1)\in J}\left\{X_{i1}^T(\bar{\theta}_K - \theta^*)\right\}^2 \leqslant C_L\left\|\bar{\theta}_K - \theta^*\right\|_2^2\right\},$$

$$\mathcal{E}_5 = \left\{\frac{3}{Kn}\sum_{(k,i)\in K}\left\{X_{ki}^T(\bar{\theta}_K - \theta^*)\right\}^2 \leqslant C_L\left\|\bar{\theta}_K - \theta^*\right\|_2^2\right\}.$$

For event $\mathcal{E}_1$, it is a result of lemma 8. While for $\mathcal{E}_2, \mathcal{E}_3$, we can apply lemma 12 and union bound. For $\mathcal{E}_4, \mathcal{E}_5$, they are the result from lemma 16. Plugging in the results above, it can be obtained that: $\mathbb{P}\left(\bigcap_{i=1}^5 \mathcal{E}_i\right) \geqslant 1 - \frac{M}{(p\vee n)^8} - \frac{M}{n^8}$.

Constrain $\|\delta\|_2 \leqslant C_1\sqrt{\frac{s_1 \log(p\vee n)}{n}}$, we can see that for the loss function of our DisC^{2}o-HD estimator satisfies

$$
\begin{aligned}
&\tilde{Q}(\theta^* + \delta) - \tilde{Q}(\theta^*) - \nabla\tilde{Q}(\theta^*)\delta \\
&= Q_1(\theta^* + \delta) - Q_1(\theta^*) - \nabla Q_1(\theta^*)\delta + \frac{1}{2}\delta^T\left(\nabla^2 Q_N(\bar{\theta}_K) - \nabla^2 Q_1(\bar{\theta}_K)\right)\delta \\
&= Q_1(\theta^* + \delta) - Q_1(\theta^*) - \nabla Q_1(\theta^*)\delta - \frac{1}{2}\delta^T\nabla^2 Q_1(\theta^*)\delta + \frac{1}{2}\delta^T\nabla^2 Q_N(\bar{\theta}_K)\delta + \frac{1}{2}\delta^T\left(\nabla^2 Q_1(\theta^*) - \nabla^2 Q_1(\bar{\theta}_K)\right)\delta \\
&= o(\|\delta\|_2^2) + \frac{1}{2}\delta^T\nabla^2 Q_N(\bar{\theta}_K)\delta + \frac{1}{2}\delta^T\left(\nabla^2 Q_1(\theta^*) - \nabla^2 Q_1(\bar{\theta}_K)\right)\delta \\
&\geqslant \mu\|\delta\|_2^2 - \mu'\frac{\log p}{n}\|\delta\|_1^2 + \frac{1}{2}\delta^T\left(\nabla^2 Q_1(\theta^*) - \nabla^2 Q_1(\bar{\theta}_K)\right)\delta + o(\|\delta\|_2^2),
\end{aligned}
$$

where the last inequality is a direct result of lemma 10 and algebra. In addition,

$$
\left|\frac{1}{2}\delta^T\left(\nabla^2 Q_1(\theta^*) - \nabla^2 Q_1(\bar{\theta}_K)\right)\delta\right| = \left|\frac{1}{n}\sum_{(i,1)\in J}\exp\left(-X_{i1}^T(\theta^* + t(\bar{\theta}_K - \theta^*))\right)X_{i1}^T(\bar{\theta}_K - \theta^*)\left(X_{i1}^T\delta\right)^2\right| = o(\|\delta\|_2^2).
$$

Then, we have

$$
\tilde{Q}(\theta^* + \delta) - \tilde{Q}(\theta^*) - \nabla\tilde{Q}(\theta^*)\delta \geqslant \mu\|\delta\|_2^2 - \mu'\frac{\log p}{n}\|\delta\|_1^2 + o(\|\delta\|_2^2) \geqslant \frac{\mu}{2}\|\delta\|_2^2 - \mu'\frac{\log p}{n}\|\delta\|_1^2.
$$

Thus, $\tilde{Q}$ also satisfies the RSC condition given in Negahban et al. (2012). Then, by directly applying Corollary 1 of Negahban et al. (2012), we have:

$$
\left\|\tilde{\theta}_K - \theta^*\right\|_2 \leqslant \frac{3\sqrt{s_1}\lambda_{\text{ps}}}{C},
$$

for every $\lambda_{\text{ps}} \geqslant \left\|\nabla\tilde{Q}(\theta^*)\right\|_\infty$.

We can see that

$$
\begin{aligned}
\nabla\tilde{Q}(\theta^*) &= \nabla Q_1(\theta^*) + (\nabla Q_N(\bar{\theta}_K) - \nabla Q_1(\bar{\theta}_K)) + (\nabla^2 Q_N(\bar{\theta}_K) - \nabla^2 Q_1(\bar{\theta}_K))(\theta^* - \bar{\theta}_K) \\
&= \nabla Q_1(\theta^*) - \nabla Q_1(\bar{\theta}_K) + \nabla Q_N(\bar{\theta}_K) - \nabla Q_N(\theta^*) + \nabla Q_N(\theta^*) + (\nabla^2 Q_N(\bar{\theta}_K) - \nabla^2 Q_1(\bar{\theta}_K))(\theta^* - \bar{\theta}_K) \\
&= \nabla Q_N(\theta^*) + \left(\nabla^2 Q_1(\bar{\theta}_K) - \nabla^2 Q_1(\theta^* + t_1(\bar{\theta}_K - \theta^*))\right)(\bar{\theta}_K - \theta^*) \\
&+ \left(\nabla^2 Q_N(\theta^* + t_2(\bar{\theta}_K - \theta^*)) - \nabla^2 Q_N(\bar{\theta}_K)\right)(\bar{\theta}_K - \theta^*),
\end{aligned}
$$

where $t_1, t_2 \in [0, 1]$. Under $\mathcal{E}_4 \cap \mathcal{E}_5$, we can see:

$$
\begin{aligned}
\left\|\left(\nabla^2 Q_1(\bar{\theta}_K) - \nabla^2 Q_1(\theta^* + t_1(\bar{\theta}_K - \theta^*))\right)(\bar{\theta}_K - \theta^*)\right\|_\infty &\leqslant t_1\left\|\frac{1}{n}\sum_{(i,1)\in J}\exp\left(-X_{i1}^T(\theta^* + s(\bar{\theta}_K - \theta^*))\right)X_{i1}\left(X_{i1}^T(\bar{\theta}_K - \theta^*)\right)^2\right\|_\infty \\
&\leqslant M\|X_{i1}\|_\infty\left|\frac{1}{n}\sum_{(1,j)\in J}\left\{X_{i1}^T(\bar{\theta}_K - \theta^*)\right\}^2\right| \\
&\leqslant MC\log(p\vee n)\left\|\bar{\theta}_K - \theta^*\right\|_2^2.
\end{aligned}
$$

Notice that we can deal with the last term in a similar manner. Plugging it back in the equation we have, it shall be observed that:

$$
\left\|\nabla\tilde{Q}(\theta^*)\right\|_\infty \leqslant \|\nabla Q_N(\theta^*)\|_\infty + 2M'\log(p\vee n)\left\|\bar{\theta}_K - \theta^*\right\|_2^2.
$$

Under event $\bigcap_{i=0}^{6} \mathcal{E}_i$, we can see that:

$$\left\|\nabla \tilde{Q}(\theta^*)\right\|_\infty \leqslant C_1 \sqrt{\frac{\log(p \vee Kn)}{Kn}} + C_2 \frac{s_1 \log^2(p \vee n)}{n}.$$

Then, with properly chosen λ_{ps}, under $\bigcap_{i=0}^{6} \mathcal{E}_i$, we can see that:

$$\left\|\tilde{\theta}_K - \theta^*\right\|_2 \leqslant C_L \left(\sqrt{\frac{s_1 \log(p \vee Kn)}{Kn}} + \frac{s_1^{3/2} \log^2(p \vee n)}{n} \right).$$

Second Claim:

Let $S = \{i : \theta^*_{Ji} \neq 0\}$. By definition, we can see that:

$$\tilde{Q}(\tilde{\theta}_K) + \lambda_{\text{ps}} \left\|\tilde{\theta}_K\right\|_1 \leqslant \tilde{Q}(\theta^*) + \lambda_{\text{ps}} \left\|\theta^*\right\|_1, \tag{1}$$
$$\tilde{Q}(\tilde{\theta}_K) - \tilde{Q}(\theta^*) - \nabla \tilde{Q}(\theta^*)(\tilde{\theta}_K - \theta^*) + \left\|\tilde{\theta}_{JS^c}\right\|_1 \leqslant -\nabla \tilde{Q}(\theta^*)(\tilde{\theta}_K - \theta^*) + \lambda_{\text{ps}} \left(\left\|(\theta^* - \tilde{\theta}_K)_S\right\|_1 \right).$$

On the left hand side, we can see that

$$\begin{aligned}
&\tilde{Q}(\tilde{\theta}_K) - \tilde{Q}(\theta^*) - \nabla \tilde{Q}(\theta^*)(\tilde{\theta}_K - \theta^*) \\
&= \left(\nabla \tilde{Q}(\theta^* + t(\tilde{\theta}_K - \theta^*)) - \nabla \tilde{Q}(\theta^*) \right) (\tilde{\theta}_K - \theta^*) \\
&= t(\tilde{\theta}_K - \theta^*)^T \left(\nabla^2 \tilde{Q}(\theta^* + t'(\tilde{\theta}_K - \theta^*)) \right) (\tilde{\theta}_K - \theta^*). \\
&= t(\tilde{\theta}_K - \theta^*)^T \left(\nabla^2 Q_1(\theta^* + t'(\tilde{\theta}_K - \theta^*)) + \nabla^2 Q_N(\bar{\theta}_K) - \nabla^2 Q_1(\bar{\theta}_K) \right) (\tilde{\theta}_K - \theta^*). \\
&= t \frac{3}{Kn} \sum_{(k,i) \in K} \left\{ \exp(-X_{ki}^T \bar{\theta}_K) \left(X_{ki}^T (\tilde{\theta}_K - \theta^*) \right)^2 + O\left(\left(X_{ki}^T (\tilde{\theta}_K - \theta^*) \right)^2 \left(X_{ki}^T (\bar{\theta}_K - \theta^*) \right) \right) \right\},
\end{aligned} \tag{2}$$

where $t, t' \in [0, 1]$.

Furthermore, under assumption 1, the left hand side satisfies:

$$\tilde{Q}(\tilde{\theta}_K) - \tilde{Q}(\theta^*) - \nabla \tilde{Q}(\theta^*)(\tilde{\theta}_K - \theta^*) \geqslant C \frac{3}{Kn} \sum_{(k,i) \in K} \left\{ X_{ki}^T (\tilde{\theta}_K - \theta^*) \right\}^2.$$

On the right hand side, under $\sum_{i=0}^{6} \mathcal{E}_i$, we have:

$$\begin{aligned}
\tilde{Q}(\theta^*)(\theta^* - \tilde{\theta}_K) + \lambda_{\text{ps}} \left(\left\|(\theta^* - \tilde{\theta}_K)_S\right\|_1 \right) &\leqslant \left\|\nabla \tilde{Q}(\theta^*)\right\|_\infty \left\|\theta^* - \tilde{\theta}_K\right\|_1 + \lambda_{\text{ps}} \left\|\theta^* - \tilde{\theta}_K\right\|_1 \\
&\leqslant C_L \left(\frac{s_1 \log(p \vee Kn)}{Kn} + \frac{s_1^3 \log^4(p \vee n)}{n^2} \right).
\end{aligned}$$

Plugging in the result we have on left hand side, the desired result is thus obtained.

Third Claim:
By equation (2), we can see that under $\bigcap_{i=0}^{6} \mathcal{E}_i$,

$$\tilde{Q}(\tilde{\theta}_K) - \tilde{Q}(\theta^*) \geqslant \nabla \tilde{Q}(\theta^*)(\tilde{\theta}_K - \theta^*).$$

Since

$$|\nabla \tilde{Q}(\theta^*)(\tilde{\theta}_K - \theta^*)| \leqslant \left\|\nabla \tilde{Q}(\theta^*)\right\|_\infty \|\tilde{\theta}_K - \theta^*\|_1,$$

we can plug these facts in (1). Thus,

$$-\left(\left\|\nabla\tilde{Q}(\theta^*)\right\|_\infty \|\tilde{\theta}_K-\theta^*\|_1\right)+\lambda_{\text{ps}}\left\|\tilde{\theta}_K\right\|_1 \leqslant \lambda_{\text{ps}}\left\|\theta^*\right\|_1.$$

Denote $\frac{\lambda_{\text{ps}}}{\left\|\nabla\tilde{Q}(\theta^*)\right\|_\infty}=c$, we have $\left\|\nabla\tilde{Q}(\theta^*)\right\|_\infty = c\lambda_{\text{ps}}$. Since $\|\tilde{\theta}_K\|_1 = \left\|\theta^*+(\tilde{\theta}_K-\theta^*)_{S^c}+(\tilde{\theta}_K-\theta^*)_S\right\| = \left\|\theta^*+(\tilde{\theta}_K-\theta^*)_{S^c}\right\|_1+\left\|(\tilde{\theta}_K-\theta^*)_S\right\|_1$, we can see that:

$$-c\lambda_{\text{ps}}\|\tilde{\theta}_K-\theta^*\|_1+\lambda_{\text{ps}}\left\|(\tilde{\theta}_K-\theta^*)_S\right\|_1 \leqslant \lambda_{\text{ps}}\left(\|\theta^*\|_1-\left\|\theta^*+(\tilde{\theta}_K-\theta^*)_{S^c}\right\|_1\right) \leqslant \lambda_{\text{ps}}\left\|\left(\tilde{\theta}_K-\theta^*\right)_S\right\|_1.$$

That is,

$$\left\|\left(\tilde{\theta}_K-\theta^*\right)_{S^c}\right\|_1 \leqslant \frac{1+c}{1-c}\left\|\left(\tilde{\theta}_K-\theta^*\right)_S\right\|_1.$$

Thus, under $\bigcap_{i=0}^6 \mathcal{E}_i$,

$$\frac{3}{Kn}\sum_{(i,j)\in K^c}\left\{X_{ki}^T(\tilde{\theta}_K-\theta^*)\right\}^2 \leqslant C_L\left(\frac{s_1\log(p\vee Kn)}{Kn}+\frac{s_1^3\log^4(p\vee n)}{n^2}\right).$$

Since $\lambda_{\text{ps}} \geqslant \left\|\nabla\tilde{Q}(\theta^*)\right\|_\infty$, $c\leqslant 1$. Thus, by taking $\lambda_{\text{ps}}=2\left\|\nabla\tilde{Q}(\theta^*)\right\|_\infty$, we can apply lemma 6 of Bradic et al. (2019), and the desired result is obtained. □

Notice that the same result should still hold if we interchange J with K^c. Thus, this result is equivalent to proposition 1.

Proposition 2. *Under assumption 1-6, with* $\lambda_{om} \asymp \sqrt{\frac{\log(p\vee Kn)}{Kn}}$*, for DisC^{2}o-HD estimator* $\tilde{\beta}_K$*, we have:*

$$\left\|\tilde{\beta}_K-\beta^*\right\|_2 \leqslant C_L\left(\sqrt{\frac{s_2\log(p\vee Kn)}{Kn}}\right),$$
$$\frac{3}{Kn}\sum_{(i,j)\in K}\left\{X_{ki}^T(\tilde{\beta}_K-\beta^*)\right\}^2 \leqslant C_L\left(\frac{s_2\log(p\vee Kn)}{Kn}\right),$$
$$\frac{3}{Kn}\sum_{(i,j)\in K^c}\left\{X_{ki}^T(\tilde{\beta}_K-\beta^*)\right\}^2 \leqslant C_L\left(\frac{s_2\log(p\vee Kn)}{Kn}\right).$$

holds with probability at least $1-\frac{M}{(p\vee n)^8}-\frac{M}{n^8}$*, where* $K=K_1$*,* K_2*, or* K_3 *and* K^c *represents another set of servers.*

Proof. **First Claim:**

Consider the events defined as:

$$\mathcal{E}_1=\left\{\left\|\nabla^2 L_N(\beta^*,\tilde{\theta}_{K^c})-\nabla^2 L_1(\beta^*,\tilde{\theta}_{K^c})\right\|_\infty \leqslant C_1\sqrt{\frac{\log(p\vee n)}{n}}\right\},$$
$$\mathcal{E}_2=\left\{\left\|\nabla L_N(\beta^*,\tilde{\theta}_{K^c})\right\|_\infty \leqslant C_1\sqrt{\frac{\log(p\vee Kn)}{Kn}}\right\}.$$

For $\mathcal{E}_1,\mathcal{E}_2$, we can apply lemma 12 and union bound, where we can consider $\tilde{\theta}_{K^c}$ to be fixed. Combining the results above, we can see that $\mathbb{P}\left(\bigcap_{i=1}^2\mathcal{E}_i\right)\geqslant 1-\frac{M}{(p\vee n)^8}$.

We can see that for the loss function of our DisC^{2}o-HD estimator, we have,

$$\begin{aligned}
&\tilde{L}(\beta^* + \delta, \tilde{\theta}_{K^c}) - \tilde{L}(\beta^*, \tilde{\theta}_{K^c}) - \nabla \tilde{L}(\beta^*, \tilde{\theta}_{K^c})^T \delta \\
&= L_1(\beta^* + \delta, \tilde{\theta}_{K^c}) - L_1(\beta^*, \tilde{\theta}_{K^c}) - \nabla L_1(\beta^*, \tilde{\theta}_{K^c})^T \delta + \frac{1}{2}\delta^T \left(\nabla^2 L_N(\bar{\beta}_K, \tilde{\theta}_{K^c}) - \nabla^2 L_1(\bar{\beta}_K, \tilde{\theta}_{K^c})\right)\delta. \\
&= \frac{1}{2}\delta^T \nabla^2 L_1(\bar{\beta}_K, \tilde{\theta}_{K^c})\delta + \frac{1}{2}\delta^T \left(\nabla^2 L_N(\bar{\beta}_K, \tilde{\theta}_{K^c}) - \nabla^2 L_1(\bar{\beta}_K, \tilde{\theta}_{K^c})\right)\delta \\
&= \frac{1}{2}\delta^T \nabla^2 L_N(\bar{\beta}_K, \tilde{\theta}_{K^c})\delta \\
&\geqslant C\|\delta\|_2^2,
\end{aligned}$$

where the last inequality is a direct result of lemma 9 and algebra. Thus, $\tilde{L}$ also satisfies the RSC condition given in Negahban et al. (2012). Then, by directly applying Corollary 1 of Negahban et al. (2012), we have:

$$\left\|\tilde{\beta}_K - \beta^*\right\|_2 \leqslant \frac{3\sqrt{s_2}\lambda_{\text{om}}}{C},$$

for every $\lambda_{\text{om}} \geqslant \left\|\nabla \tilde{L}(\beta^*, \tilde{\theta}_{K^c})\right\|_\infty$.

Following the same technique as proposition 1, we can show that

$$\begin{aligned}
&\nabla \tilde{L}(\beta^*, \tilde{\theta}_{K^c}) \\
&= \nabla L_1(\beta^*, \tilde{\theta}_{K^c}) + (\nabla L_N(\bar{\beta}_K, \tilde{\theta}_{K^c}) - \nabla L_1(\bar{\beta}_K, \tilde{\theta}_{K^c})) + \left(\nabla^2 L_N(\bar{\beta}_K, \tilde{\theta}_{K^c}) - \nabla^2 L_1(\bar{\beta}_K, \tilde{\theta}_{K^c})\right)(\beta^* - \bar{\beta}_K) \\
&= \nabla L_1(\beta^*, \tilde{\theta}_{K^c}) - \nabla L_1(\bar{\beta}_K, \tilde{\theta}_{K^c}) + \nabla L_N(\bar{\beta}_K, \tilde{\theta}_{K^c}) - \nabla L_N(\beta^*, \tilde{\theta}_{K^c}) + \nabla L_N(\beta^*, \tilde{\theta}_{K^c}) \\
&+ \left(\nabla^2 L_N(\bar{\beta}_K, \tilde{\theta}_{K^c}) - \nabla^2 L_1(\bar{\beta}_K, \tilde{\theta}_{K^c})\right)(\beta^* - \bar{\beta}_K) \\
&= \left(\nabla^2 L_N(\beta^*, \tilde{\theta}_{K^c}) - \nabla^2 L_N(\bar{\beta}_K, \tilde{\theta}_{K^c})\right)(\bar{\beta}_K - \beta^*) - \left(\nabla^2 L_1(\beta^*, \tilde{\theta}_{K^c}) - \nabla^2 L_1(\bar{\beta}_K, \tilde{\theta}_{K^c})\right)(\bar{\beta}_K - \beta^*) + \nabla L_N(\beta^*, \tilde{\theta}_{K^c}) \\
&= \nabla L_N(\beta^*, \tilde{\theta}_{K^c}).
\end{aligned}$$

Then, we can see that under $\bigcap_{i=1}^2 \mathcal{E}_i$

$$\left\|\nabla \tilde{L}(\beta^*, \tilde{\theta}_{K^c})\right\|_\infty \leqslant \left\|\nabla L_N(\beta^*, \tilde{\theta}_{K^c})\right\|_\infty.$$

Under event $\bigcap_{i=1}^2 \mathcal{E}_i$, we can see that:

$$\left\|\nabla \tilde{L}(\beta^*, \tilde{\theta}_{K^c})\right\|_\infty \leqslant C_1\sqrt{\frac{\log(p \vee Kn)}{Kn}}.$$

Then, with properly chosen λ_{om}, under $\bigcap_{i=1}^2 \mathcal{E}_i$, we can see that:

$$\left\|\tilde{\beta}_K - \beta^*\right\|_2 \leqslant C_L\sqrt{\frac{s_2\log(p \vee Kn)}{Kn}}.$$

Second and Third Claim:
The proof is an analog of proposition 1. □

Notice that the same result still holds if we interchange J with K^c. Thus, this result is equivalent to lemma 2.

Theorem 3. *Under assumption 1-6, the distributed estimator for DisC^{2}o-HD method satisfies*

$$|\tilde{\tau}_1 - \hat{\tau}_1^*| \leqslant C_L\left(\frac{\sqrt{s_2(s_1 \vee s_2)}\log(p \vee Kn)}{Kn} + \frac{\sqrt{s_2(s_1 \vee s_2)\log(p \vee Kn)\log^4(p \vee n)}}{n\sqrt{Kn}}\right)$$

with probability at least $1 - \frac{M}{(p\vee n)^8} - \frac{M}{n^8}$, where C_L is a sufficiently large constant and M is another constant depending on C_L.

Proof. We focus on $\left|\tilde{\tau}_{1,K_1} - \hat{\tau}^*_{1,K_1}\right|$ first. While $\left|\tilde{\tau}_{1,K_2} - \hat{\tau}^*_{1,K_2}\right|$ and $\left|\tilde{\tau}_{1,K_3} - \hat{\tau}^*_{1,K_3}\right|$ can be dealt with in a similar manner. Consider the events defined as:

$$\begin{aligned}
\mathcal{E}_0 &= \left\{ \|\tilde{\theta}_{K^c} - \theta^*\|_2 \leqslant C_L \left(\sqrt{\frac{s_1 \log(p \vee Kn)}{Kn}} + \frac{s_1^{3/2} \log^2(p \vee n)}{n} \right) \right\}, \\
\mathcal{E}_1 &= \left\{ \|\tilde{\beta}_{K^c} - \beta^*\|_2 \leqslant C_L \sqrt{\frac{s_2 \log(p \vee Kn)}{Kn}} \right\}, \\
\mathcal{E}_2 &= \Bigg\{ \frac{3}{Kn} \sum_{(k,i)\in K} \left\{ X_{ki}^T(\tilde{\theta}_{K^c} - \theta^*) \right\}^2 \leqslant C_L \left(\frac{s_1 \log(p \vee Kn)}{Kn} + \frac{s_1^3 \log^4(p \vee n)}{n^2} \right), \\
&\qquad \frac{3}{Kn} \sum_{(k,i)\in K} \left\{ X_{ki}^T(\tilde{\beta}_{K^c} - \beta^*) \right\}^2 \leqslant C_L \frac{s_2 \log(p \vee Kn)}{Kn} \Bigg\}, \\
\mathcal{E}_3 &= \left\{ \left\| \frac{3}{Kn} \sum_{(k,i)\in K} \left\{ \left(\frac{T_{ki}}{\pi(X_{ki}^T \theta^*)} - 1 \right) X_{ki}^T \right\} \right\|_\infty \leqslant C_L \sqrt{\frac{\log(p \vee Kn)}{Kn}} \right\}, \\
\mathcal{E}_4 &= \left\{ \left\| \frac{3}{Kn} \sum_{(k,i)\in K} X_{ki} \varepsilon_{ki}^* \right\|_\infty \leqslant C_L \sqrt{\frac{\log(p \vee Kn)}{Kn}} \right\}.
\end{aligned}$$

By proposition 1 and 2 we may realize that $\mathcal{E}_0, \mathcal{E}_1, \mathcal{E}_2$ will hold with probability at least $1 - \frac{M}{(p \vee n)^8} - \frac{M}{n^8}$ for both one-step and DisC^{2}o-HD estimator. We can see that $\frac{T_{ki}}{\pi(X_{ki}^T \theta^*)} - 1$ is bounded by the strong ignorability assumption, with zero expected value. By lemma 12 and union bound, we can see event $\mathcal{E}_3, \mathcal{E}_4$ will hold with probability at least $1 - \frac{M}{(p \vee Kn)^8}$. Thus, $\bigcap_{i=0}^4 \mathcal{E}_i$ will hold with probability at least $1 - \frac{M}{(p \vee n)^8} - \frac{M}{n^8}$.

By rearranging the terms, we have:

$$\begin{aligned}
\hat{\tau}^*_{1,J} - \tilde{\tau}_{1,J} &= \frac{3}{Kn} \sum_{(k,i)\in K} \left\{ X_{ki}^T \beta^* + \frac{T_{ki}}{\pi(X_{ki}^T \theta^*)} \left(Y_{ki} - X_{ki}^T \beta^* \right) \right\} - \frac{3}{Kn} \sum_{(k,i)\in K} \left\{ \left(X_{ki}^T \tilde{\beta}_{K^c} + \frac{T_{ki}}{\pi(X_{ki}^T \tilde{\theta}_{K^c})} \left(Y_{ki} - X_{ki}^T \tilde{\beta}_{K^c} \right) \right) \right\} \\
&= \Delta_1 + \Delta_2 + \Delta_3.
\end{aligned}$$

where

$$\begin{aligned}
\Delta_1 &= \frac{3}{Kn} \sum_{(k,i)\in K} \left(\frac{T_{ki}}{\pi\left(X_{ki}^T \tilde{\theta}_{K^c}\right)} - \frac{T_{ki}}{\pi\left(X_{ki}^T \theta^*\right)} \right) X_{ki}^T \left(\tilde{\beta}_{K^c} - \beta^* \right), \\
\Delta_2 &= \frac{3}{Kn} \sum_{(k,i)\in K} \left(\frac{T_{ki}}{\pi\left(X_{ki}^T \theta^*\right)} - 1 \right) X_{ki}^T \left(\tilde{\beta}_{K^c} - \beta^* \right), \\
\Delta_3 &= \frac{3}{Kn} \sum_{(k,i)\in K} \left(\frac{T_{ki}}{\pi(X_{ki}^T \theta^*)} - \frac{T_{ki}}{\pi(X_{ki}^T \tilde{\theta}_{K^c})} \right) \left(Y_{ki} - X_{ki}^T \beta^* \right).
\end{aligned}$$

Consider Δ_1, we may see that

$$\begin{aligned}
|\Delta_1| &= \left| \frac{3}{Kn} \sum_{(k,i)\in K} T_{ki} \left\{ \left(\exp\left(-X_{ki}^T \tilde{\theta}_{K^c}\right) - \exp\left(-X_{ki}^T \theta^*\right) \right) X_{ki}^T \left(\tilde{\beta}_{K^c} - \beta^* \right) \right\} \right| \\
&\leqslant C' \left(\frac{3}{Kn} \sum_{(k,i)\in K} \left\{ X_{ki}^T \left(\tilde{\beta}_{K^c} - \beta^* \right) \right\}^2 \right)^{1/2} \left(\frac{3}{Kn} \sum_{(k,i)\in K} \left\{ X_{ki}^T \left(\tilde{\theta}_{K^c} - \theta^* \right) \right\}^2 \right)^{1/2},
\end{aligned}$$

where we applied the mean value theorem and Cauchy inequality in the second line. Under $\mathcal{E}_0 \bigcap \mathcal{E}_1$, we have :

$$|\Delta_1| \leqslant C\left(\frac{\sqrt{s_1 s_2}\log(p \vee Kn)}{Kn} + \frac{s_1\sqrt{s_1 s_2 \log(p \vee Kn)\log^4(p \vee n)}}{n\sqrt{Kn}}\right).$$

While for Δ_2, we have:

$$\begin{aligned}
\Delta_2 &= \frac{3}{Kn}\sum_{(k,i)\in K}\left\{\left(\frac{T_{ki}}{\pi(X_{ki}^T\theta^*)} - 1\right)X_{ki}^T\left(\tilde{\beta}_{K^c} - \beta^*\right)\right\} \\
&\leqslant \left\|\frac{3}{Kn}\sum_{(k,i)\in K}\left\{\left(\frac{T_{ki}}{\pi(X_{ki}^T\theta^*)} - 1\right)X_{ki}^T\right\}\right\|_\infty \left\|\tilde{\beta}_{K^c} - \beta^*\right\|_1. \\
&\leqslant \left\|\frac{3}{Kn}\sum_{(k,i)\in K}\left\{\left(\frac{T_{ki}}{\pi(X_{ki}^T\theta^*)} - 1\right)X_{ki}^T\right\}\right\|_\infty \sqrt{s_2}\left\|\tilde{\beta}_{K^c} - \beta^*\right\|_2
\end{aligned}$$

Thus, under $\mathcal{E}_1 \bigcap \mathcal{E}_3$,

$$|\Delta_2| \leqslant C_L \frac{s_2 \log(p \vee Kn)}{Kn}.$$

While for Δ_3, we can take advantage of the sample splitting method, which would give us:

$$\begin{aligned}
&\mathbb{E}\left[\frac{3}{Kn}\sum_{(k,i)\in K}\left\{\left(\frac{T_{ki}}{\pi(X_{ki}^T\tilde{\theta}_{K^c})} - \frac{T_{ki}}{\pi(X_{ki}^T\theta^*)}\right)\right\}\varepsilon_{ki}^*\right] \\
&= \mathbb{E}\left[\mathbb{E}\left[\frac{3}{Kn}\sum_{(k,i)\in K}\left(\frac{T_{ki}}{\pi(X_{ki}^T\tilde{\theta}_{K^c})} - \frac{T_{ki}}{\pi(X_{ki}^T\theta^*)}\right)\varepsilon_{ki}^*\middle|\{X_{ki}, Y_{ki}, T_{ki}\}_{(i,j)\in K^c}\bigcap\{X_{ki}\}_{(k,i)\in K}\right]\right] = 0
\end{aligned}$$

Notice that if we condition on $\{X_{ki}, Y_{ki}, T_{ki}\}_{i\in J_K^c}\bigcap\{X_{ki}\}_K$, $\pi(X_{ki}^T\tilde{\theta}_{K^c})$ can be considered as fixed.

Then, we can consider the truncated case, where $\mathcal{E}_5 = \left\{\frac{3}{Kn}\sum_{(k,i)\in K}\left(\frac{T_{ki}}{\pi(X_{ki}^T\tilde{\theta}_{K^c})} - \frac{T_{ki}}{\pi(X_{ki}^T\theta^*)}\right)\varepsilon_{ki}^* \geqslant t\right\}$ for some t to be chosen later. In the meanwhile, we can denote:

$$A = \left\{\frac{3}{Kn}\sum_{(k,i)\in K}\left\{X_{ki}^T\left(\tilde{\theta}_{K^c} - \theta^*\right)\right\}^2 \leqslant C_L\left(\frac{s_1\log(p \vee Kn)}{Kn} + \frac{s_1^3\log^2(p \vee n)}{n^2}\right)\right\}.$$

Then, we can see that:

$$\begin{aligned}
\mathbb{P}(\mathcal{E}_5) &= \mathbb{E}\left[\mathbb{P}\left(\mathcal{E}_5\middle|\{T_{ki}, X_{ki}, Y_{ki}\}_{K^c}\bigcap\{X_{ki}\}_K\right)\right] \\
&= \mathbb{E}\left[\mathbb{P}\left(\mathcal{E}_5\middle|\{T_{ki}, X_{ki}, Y_{ki}\}_{K^c}\bigcap\{X_{ki}\}_K\right)\mathbb{1}\{A\}\right] + \mathbb{E}\left[\mathbb{P}\left(\mathcal{E}_5\middle|\{T_{ki}, X_{ki}, Y_{ki}\}_{K^c}\bigcap\{X_{ki}\}_K\right)\mathbb{1}\{A^c\}\right].
\end{aligned}$$

Denote $\Delta_{Kn}^2 = \frac{3}{Kn}\sum_{(k,i)\in K}\left\{\left(\exp(-X_{ki}^T\theta^*) - \exp(-X_{ki}^T\tilde{\theta}_{K^c})\right)\right\}^2$. Since $T_{ki}\varepsilon_{ki}^*$ are sub-Gaussian, by assumption 4

and the fact that $T_{ki} \in \{0,1\}$, by Hoeffding inequality, we have

$$\begin{aligned}
&\mathbb{E}\left(\exp\left(-\frac{CKnt^2}{\Delta_{Kn}^2}\right)\right) \\
&\leqslant \mathbb{E}\left(\exp\left(-\frac{CKnt^2}{\Delta_{Kn}^2}\right)\mathbb{1}\left\{\frac{3}{Kn}\sum_{(k,i)\in K}\left\{X_{ki}^T\left(\tilde{\theta}_{K^c}-\theta^*\right)\right\}^2 \leqslant C_L\left(\frac{s_1\log(p\vee Kn)}{Kn}+\frac{s_1^3\log^4(p\vee n)}{n^2}\right)\right\}\right) \\
&+\mathbb{P}\left(A^c\right) \\
&\leqslant \mathbb{E}\left(\exp\left(-\frac{CKnt^2}{C_L\left(\frac{s_1\log(p\vee Kn)}{Kn}+\frac{s_1^3\log^4(p\vee n)}{n^2}\right)}\right)\right)+\mathbb{P}\left(A^c\right).
\end{aligned}$$

Taking $t^2 = C_L' \frac{\log(p\vee Kn)}{Kn}\left(\frac{s_1\log(p\vee Kn)}{Kn}+\frac{s_1^3\log^4(p\vee n)}{n^2}\right)$:

$$\begin{aligned}
\mathbb{P}\left(\mathcal{E}_5\right) &= \mathbb{P}\left(|\Delta_3| \geqslant C_L\sqrt{\frac{\log(p\vee Kn)}{Kn}}\left(\sqrt{\frac{s_1\log(p\vee Kn)}{Kn}}+\frac{s_1^{3/2}\log^2(p\vee n)}{n}\right)\right) \\
&\leqslant \frac{M}{(p\vee Kn)^8}.
\end{aligned}$$

Thus, we can see that event

$$|\Delta_3| \leqslant \sqrt{\frac{\log(p\vee Kn)}{Kn}}\left(\sqrt{\frac{s_1\log(p\vee Kn)}{Kn}}+\frac{s_1^{3/2}\log^2(p\vee n)}{n}\right)$$

will hold with probability at least $1-\frac{M}{(p\vee Kn)^8}$. Combining the event and probability we obtained at Δ_1, Δ_2, and Δ_3, we have

$$\begin{aligned}
&\mathbb{P}\left(|\tilde{\tau}_{1,J}-\hat{\tau}_{1,J}^*| \leqslant C_L\left(\frac{\sqrt{s_2(s_1\vee s_2)}\log(p\vee Kn)}{Kn}+\frac{s_1\sqrt{s_1s_2\log(p\vee Kn)\log^4(p\vee n)}}{n\sqrt{Kn}}\right)\right) \\
&\geqslant 1-\frac{M}{(p\vee n)^8}-\frac{M}{n^8}.
\end{aligned}$$

Likewise, we can apply the same technique to $\tilde{\tau}_{1,K_2}$ or $\tilde{\tau}_{1,K_3}$. Combining the bound above, the desired bound can be obtained. □

Theorem 4. *Under assumptions 1-6, we have*

$$\begin{aligned}
&\sup_{x\in\mathbb{R}}\left|\mathbb{P}\left(\frac{\sqrt{Kn}(\tilde{\tau}_1-\tau_1^*)}{\sqrt{\widehat{V}}} \leqslant x\right)-\Phi(x)\right| \\
&\leqslant \frac{M}{(p\vee n)^8}+\frac{M}{n^8}+C_L\left(\frac{\sqrt{s_2(s_1\vee s_2)}\log(p\vee Kn)}{\sqrt{Kn}}+\frac{s_1\sqrt{s_1s_2\log(p\vee Kn)\log^4(p\vee n)}}{n}\right),
\end{aligned}$$

where C_L is a sufficiently large constant, and M depends on C_L.

Proof. We prove this theorem in two steps. Firstly, we control

$$\sup_{x\in\mathbb{R}}\left|\mathbb{P}\left(\frac{\sqrt{Kn}(\tilde{\tau}_1-\tau_1^*)}{\sqrt{V^*}} \leqslant x\right)-\Phi(x)\right|.$$

Then, with the Berry-Esseen type bound established, we can replace the true value of the variance with the variance estimator to bound

$$\sup_{x\in\mathbb{R}}\left|\mathbb{P}\left(\frac{\sqrt{Kn}(\tilde{\tau}_1-\tau_1^*)}{\sqrt{\widehat{V}}}\leqslant x\right)-\Phi(x)\right|.$$

For the first term, consider $\hat{\tau}_1^*$ defined as:

$$\hat{\tau}_1^*:=\frac{1}{Kn}\sum_{j=1}^{m}\sum_{i=1}^{n}\left\{X_{ki}^T\beta^*+\frac{T_{ki}}{\pi(X_{ki}^T\theta^*)}\left(Y_{ki}-X_{ki}^T\beta^*\right)\right\},$$

then we can see that the classical Berry-Esseen bound holds under assumption 6:

$$\sup_{x\in\mathbb{R}}\left|\mathbb{P}\left(\frac{\sqrt{Kn}(\hat{\tau}_1^*-\tau_1^*)}{\sqrt{V^*}}\leqslant x\right)-\Phi(x)\right|\leqslant\frac{C}{\sqrt{Kn}}.$$

Then, we may consider the event $\mathcal{E}_1=\{|\tilde{\tau}_1-\hat{\tau}_1^*|\leqslant r\}$ where

$$r=C_L\left(\frac{\sqrt{s_2(s_1\vee s_2)}\log(p\vee Kn)}{Kn}+\frac{s_1\sqrt{s_1s_2\log(p\vee Kn)\log^4(p\vee n)}}{n\sqrt{Kn}}\right).$$

By theorem 3, we can realize that $\mathbb{P}(\mathcal{E}_1^c)\leqslant\frac{M}{(p\vee n)^8}+\frac{M}{n^8}$.

For the first term, we may realize that:

$$\mathbb{P}\left(\frac{\sqrt{Kn}(\tilde{\tau}_1-\tau_1^*)}{\sqrt{V^*}}\leqslant x\right)-\Phi(x)=\mathbb{P}\left(\frac{\sqrt{Kn}(\tilde{\tau}_1-\tau_1^*)}{\sqrt{V^*}}\leqslant x,\mathcal{E}_1\right)-\Phi(x)+\mathbb{P}\left(\frac{\sqrt{Kn}(\tilde{\tau}_1-\tau_1^*)}{\sqrt{V^*}}\leqslant x,\mathcal{E}_1^c\right).$$

It can be easily showed that:

$$\sup_{x\in\mathbb{R}}\mathbb{P}\left(\frac{\sqrt{Kn}(\tilde{\tau}_1-\tau_1^*)}{\sqrt{V^*}}\leqslant x,\mathcal{E}_1\right)-\Phi\left(x\right)\leqslant\frac{C}{\sqrt{Kn}}+\frac{M}{(p\vee n)^8}+\frac{M}{n^8}+C\sqrt{Kn}r,$$

where C is some positive constant. With similar argument, we can see that:

$$\sup_{x\in\mathbb{R}}\left|\mathbb{P}\left(\frac{\sqrt{Kn}(\tilde{\tau}_1-\tau_1^*)}{\sqrt{V^*}}\leqslant x\right)-\Phi\left(x\right)\right|\leqslant\frac{C}{\sqrt{Kn}}+\frac{M}{(p\vee n)^8}+\frac{M}{n^8}+C'\sqrt{Kn}r,$$

where C' is some positive constant.

By proposition 5,

$$\mathbb{P}\left(\left|\sqrt{\frac{\widehat{V}}{V^*}}-1\right|\leqslant C_L\left(\sqrt{\frac{(s_1\vee s_2)\log(p\vee Kn)}{Kn}}+\frac{s_1^{3/2}\log(p\vee n)}{n}\right)\right)\geqslant 1-\frac{M}{(p\vee n)^8}-\frac{M}{n^8}.$$

Thus, for the second result, we can apply the same technique as above. The same result shall be obtained. □

3.2 Consistency Lemmas

Proposition 5 (Consistency of variance estimator). *The variance estimator satisfies*

$$|\widehat{V}-V^*|\leqslant C_L\left(\sqrt{\frac{(s_1\vee s_2)\log(p\vee Kn)}{Kn}}+\frac{s_1^{3/2}\log^2(p\vee n)}{n}\right),$$

$$\left|\sqrt{\frac{\widehat{V}}{V^*}}-1\right|\leqslant C_L\left(\sqrt{\frac{(s_1\vee s_2)\log(p\vee Kn)}{Kn}}+\frac{s_1^{3/2}\log^2(p\vee n)}{n}\right),$$

with probability at least $1-\frac{M}{(p\vee n)^8}-\frac{M}{n^8}$, *where* C_L *is a sufficiently large constant and* M *depends on* C_L.

Proof. Consider events defined as

$$\mathcal{E}_0 = \left\{ \|\tilde{\theta} - \theta^*\|_2 \leqslant C_L \left(\sqrt{\frac{s_1 \log(p \vee Kn)}{Kn}} + \frac{s_1^{3/2} \log^2(p \vee n)}{n} \right) \right\},$$

$$\mathcal{E}_1 = \left\{ \|\tilde{\beta} - \beta^*\|_2 \leqslant C_L \sqrt{\frac{s_2 \log(p \vee Kn)}{Kn}} \right\}$$

$$\mathcal{E}_2 = \left\{ \frac{1}{Kn} \sum_{j=1}^{m} \sum_{i=1}^{n} (X_{ki}^T(\tilde{\theta} - \theta^*))^2 \leqslant C_L \left(\frac{s_1 \log(p \vee Kn)}{Kn} + \frac{s_1^3 \log^4(p \vee n)}{n^2} \right), \right.$$
$$\left. \frac{1}{Kn} \sum_{j=1}^{m} \sum_{i=1}^{n} (X_{ki}^T(\tilde{\beta} - \beta^*))^2 \leqslant C_L \frac{s_2 \log(p \vee Kn)}{Kn} \right\},$$

$$\mathcal{E}_3 = \left\{ \left\| \frac{1}{Kn} \sum_{j=1}^{m} \sum_{i=1}^{n} \left\{ \left(\frac{T_{ki}}{\pi(X_{ki}^T \theta^*)} - 1 \right) X_{ki}^T \right\} \right\|_\infty \leqslant C_L \sqrt{\frac{\log(p \vee Kn)}{Kn}} \right\},$$

$$\mathcal{E}_4 = \left\{ |\tau_1^* - \tilde{\tau}_1| \leqslant \frac{C_L'}{\sqrt{Kn}} \right\},$$

$$\mathcal{E}_5 = \left\{ \left| \frac{1}{Kn} \sum_{j=1}^{m} \sum_{i=1}^{n} \left\{ \frac{T_{ki}}{\pi(X_{ki}^T \theta^*)^2} \left(Y_{ki} - X_{ki}^T \beta^* \right)^2 \right\} - \mathbb{E}\left[\frac{1}{\pi(X_{ki}^T \theta^*)} (Y_{ki} - X_{ki}^T \beta^*)^2 \right] \right| \leqslant C_L \sqrt{\frac{\log(p \vee Kn)}{Kn}} \right\},$$

$$\mathcal{E}_6 = \left\{ \left| \frac{1}{Kn} \sum_{j=1}^{m} \sum_{i=1}^{n} (\varepsilon_{ki}^*)^4 - \mathbb{E}\left((\varepsilon_{ki}^*)^4 \right) \right| \leqslant C_L, \left\| \frac{1}{Kn} \sum_{j=1}^{m} \sum_{i=1}^{n} \varepsilon_{ki} X_{ki} \right\|_\infty \leqslant C_L \sqrt{\frac{\log(p \vee Kn)}{Kn}} \right\},$$

$$\mathcal{E}_7 = \left\{ \left| \frac{1}{Kn} \sum_{j=1}^{m} \sum_{i=1}^{n} \left\{ \left(X_{ki}^T \beta^* - \tau_1^* \right)^2 \right\} - \mathbb{E}\left(\left(X_{ki}^T \beta^* - \tau_1^* \right)^2 \right) \right| \leqslant C_L \sqrt{\frac{\log(p \vee Kn)}{Kn}} \right\}.$$

By proposition 1 and 2 we may realize that $\mathcal{E}_0, \mathcal{E}_1$ will hold with probability at least $1 - \frac{M}{(p \vee n)^8} - \frac{M}{n^8}$. Also, $\mathcal{E}_2$ will hold with probability at least $1 - \frac{M}{(p \vee Kn)^8}$, which is the result of lemma 16. For event $\mathcal{E}_3$, it is a combination of theorem 3, assumption 5, union bound, and lemma 12. We can see that $\frac{T_{ki}}{\pi(X_{ki}^T \theta^*)} - 1$ is bounded by the strong ignorability assumption, with zero expected value. By lemma 12 and union bound, we can see events $\mathcal{E}_4, \mathcal{E}_6, \mathcal{E}_7$ will hold with probability at least $1 - \frac{M}{(p \vee Kn)^8}$. We can show the second part of $\mathcal{E}_6$ by the same technique. For the first part of $\mathcal{E}_6$, it can be derived by lemma 15. Thus, $\bigcap_{i=0}^7 \mathcal{E}_i$ will hold with probability at least $1 - \frac{M}{(p \vee n)^8} - \frac{M}{n^8}$.

We can show that, under $\bigcap_{i=0}^7 \mathcal{E}_i$:

$$|\widehat{V} - \widehat{V}^*| \leqslant C_L \left(\sqrt{\frac{(s_1 \vee s_2) \log(p \vee Kn)}{Kn}} + \frac{s_1^{3/2} \log^2(p \vee n)}{n} \right),$$

where $\widehat{V}^* := \frac{1}{Kn} \sum_{j=1}^m \sum_{i=1}^n \left\{ \frac{T_{ki}}{\pi(X_{ki}^T \theta^*)^2} \left(Y_{ki} - X_{ki}^T \beta^* \right)^2 + \left(X_{ki}^T \beta^* - \tau_1^* \right)^2 \right\}$.

Then, we show that:

$$|\widehat{V}^* - V^*| \leqslant C_L' \sqrt{\frac{\log(p \vee Kn)}{Kn}}.$$

We may begin by rearranging the terms. Consider $\widehat{V} - \widehat{V}^*$, we have:

$$\widehat{V} - \widehat{V}^* = \frac{1}{Kn} \sum_{j=1}^{m} \sum_{i=1}^{n} \left\{ \frac{T_{ki}}{\pi(X_{ki}^T \tilde{\theta})^2} \left(Y_{ki} - X_{ki}^T \tilde{\beta} \right)^2 + \left(X_{ki}^T \tilde{\beta} - \tilde{\tau}_1 \right)^2 \right\}$$
$$- \frac{1}{Kn} \sum_{j=1}^{m} \sum_{i=1}^{n} \left\{ \frac{T_{ki}}{\pi(X_{ki} \theta^*)^2} \left(Y_{ki} - X_{ki}^T \beta^* \right)^2 + \left(X_{ki}^T \beta^* - \tau_1^* \right)^2 \right\}.$$

This can be decomposed as:

$$\widehat{V} - \widehat{V}^* = \Delta_1 + \Delta_2,$$

where

$$\Delta_1 = \frac{1}{Kn}\sum_{j=1}^{m}\sum_{i=1}^{n}\left\{\frac{T_{ki}}{\pi(X_{ki}^T\tilde{\theta})^2}\left(Y_{ki} - X_{ki}^T\tilde{\beta}\right)^2\right\} - \frac{1}{Kn}\sum_{j=1}^{m}\sum_{i=1}^{n}\left\{\frac{T_{ki}}{\pi(X_{ki}\theta^*)^2}\left(Y_{ki} - X_{ki}^T\beta^*\right)^2\right\},$$

$$\Delta_2 = \frac{1}{Kn}\sum_{j=1}^{m}\sum_{i=1}^{n}\left\{\left(X_{ki}^T\tilde{\beta} - \tilde{\tau}_1\right)^2\right\} - \frac{1}{Kn}\sum_{j=1}^{m}\sum_{i=1}^{n}\left\{\left(X_{ki}^T\beta^* - \tau_1^*\right)^2\right\}.$$

Then, we can decompose Δ_1:

$$\Delta_1 = \Delta_{11} + \Delta_{12},$$

where

$$\Delta_{11} = \frac{1}{Kn}\sum_{j=1}^{m}\sum_{i=1}^{n}\left\{\frac{T_{ki}}{\pi(X_{ki}\theta^*)^2}\left(\left(Y_{ki} - X_{ki}^T\tilde{\beta}\right)^2 - \left(Y_{ki} - X_{ki}^T\beta^*\right)^2\right)\right\},$$

$$\Delta_{12} = \frac{1}{Kn}\sum_{j=1}^{m}\sum_{i=1}^{n}\left\{\left(\frac{T_{ki}}{\pi(X_{ki}^T\tilde{\theta})^2} - \frac{T_{ki}}{\pi(X_{ki}^T\theta^*)^2}\right)\left(Y_{ki} - X_{ki}^T\tilde{\beta}\right)^2\right\}.$$

Then, for Δ_{11}, we can see that

$$\begin{aligned}\Delta_{11} &= \frac{2}{Kn}\sum_{j=1}^{m}\sum_{i=1}^{n}\left\{\frac{T_{ki}}{\pi(X_{ki}\theta^*)^2}\varepsilon_{ki}^*\left(X_{ki}^T(\tilde{\beta} - \beta^*)\right)\right\} + \frac{1}{Kn}\sum_{j=1}^{m}\sum_{i=1}^{n}\left\{\frac{T_{ki}}{\pi(X_{ki}\theta^*)^2}\left(X_{ki}^T(\tilde{\beta} - \beta^*)\right)^2\right\} \\ &\leqslant \left\|\frac{2}{Kn}\sum_{j=1}^{m}\sum_{i=1}^{n}\frac{T_{ki}}{\pi(X_{ki}^T\theta^*)^2}\varepsilon_{ki}^*X_{ki}^T\right\|_\infty \left\|\tilde{\beta} - \beta^*\right\|_1 + \frac{1}{Kn}\sum_{j=1}^{m}\sum_{i=1}^{n}\left\{X_{ki}^T(\tilde{\beta} - \beta^*)\right\}^2. \\ &\leqslant \left\|\frac{2}{Kn}\sum_{j=1}^{m}\sum_{i=1}^{n}\frac{T_{ki}}{\pi(X_{ki}^T\theta^*)^2}\varepsilon_{ki}^*X_{ki}^T\right\|_\infty \sqrt{s_2}\left\|\tilde{\beta} - \beta^*\right\|_2 + \frac{1}{Kn}\sum_{j=1}^{m}\sum_{i=1}^{n}\left\{X_{ki}^T(\tilde{\beta} - \beta^*)\right\}^2.\end{aligned}$$

Under $\mathcal{E}_1 \bigcap \mathcal{E}_2 \bigcap \mathcal{E}_6$:

$$\Delta_{11} \leqslant C_L \frac{s_2 \log(p \vee Kn)}{Kn}.$$

For Δ_{12}, we have:

$$\begin{aligned}\Delta_{12} = &\frac{1}{Kn}\sum_{j=1}^{m}\sum_{i=1}^{n}\left\{\left(\frac{T_{ki}}{\pi(X_{ki}^T\tilde{\theta})^2} - \frac{T_{ki}}{\pi(X_{ki}^T\theta^*)^2}\right)2\varepsilon_{ki}^*\left(X_{ki}^T\tilde{\beta} - X_{ki}^T\beta^*\right)\right\} \\ &+ \frac{1}{Kn}\sum_{j=1}^{m}\sum_{i=1}^{n}\left\{\left(\frac{T_{ki}}{\pi(X_{ki}^T\tilde{\theta})^2} - \frac{T_{ki}}{\pi(X_{ki}^T\theta^*)^2}\right)\left(X_{ki}^T\tilde{\beta} - X_{ki}^T\beta^*\right)^2\right\} \\ &+ \frac{1}{Kn}\sum_{j=1}^{m}\sum_{i=1}^{n}\left\{\left(\frac{T_{ki}}{\pi(X_{ki}^T\tilde{\theta})^2} - \frac{T_{ki}}{\pi(X_{ki}^T\theta^*)^2}\right)(\varepsilon_{ki}^*)^2\right\}.\end{aligned}$$

We can apply similar technique as above for the first two terms. While for the last one, by Cauchy inequality and mean value theorem:

$$\begin{aligned}&\frac{1}{Kn}\sum_{j=1}^{m}\sum_{i=1}^{n}\left\{\left(\frac{T_{ki}}{\pi(X_{ki}^T\tilde{\theta})^2} - \frac{T_{ki}}{\pi(X_{ki}^T\theta^*)^2}\right)(\varepsilon_{ki}^*)^2\right\} \\ \leqslant\, &C\left(\frac{1}{Kn}\sum_{j=1}^{m}\sum_{i=1}^{n}\left\{\left(\frac{T_{ki}}{\pi(X_{ki}^T\tilde{\theta})} + \frac{T_{ki}}{\pi(X_{ki}^T\theta^*)}\right)(\varepsilon_{ki}^*)^2\right\}^2\right)^{1/2}\left(\frac{1}{Kn}\sum_{j=1}^{m}\sum_{i=1}^{n}\left\{X_{ki}^T(\tilde{\theta} - \theta^*)\right\}^2\right)^{1/2}.\end{aligned}$$

Under $\mathcal{E}_0 \bigcap \mathcal{E}_2 \bigcap \mathcal{E}_5$

$$|\Delta_{12}| \leqslant C_L \left(\sqrt{\frac{(s_1 \vee s_2)\log(p \vee Kn)}{Kn}} + \frac{s_1^{3/2}\log^2(p \vee n)}{n} \right).$$

For Δ_2, we have:

$$\begin{aligned}
|\Delta_2| \leqslant 2 \Bigg(&\frac{1}{Kn}\sum_{j=1}^{m}\sum_{i=1}^{n}\left\{X_{ki}^T(\tilde{\beta}-\beta^*)\right\}^2 + (\tilde{\tau}_1 - \tau_1^*)^2 \\
&+ \left(\frac{1}{Kn}\sum_{j=1}^{m}\sum_{i=1}^{n}\left\{X_{ki}^T(\tilde{\beta}-\beta^*)\right\}^2\right)^{1/2} \left(\frac{1}{Kn}\sum_{j=1}^{m}\sum_{i=1}^{n}\left\{X_{ki}^T\beta^* - \tau_1^*\right\}^2\right)^{1/2} \\
&+ \left(\frac{1}{Kn}\sum_{j=1}^{m}\sum_{i=1}^{n}\left\{X_{ki}^T\beta^* - \tau_1^*\right\}^2\right)^{1/2} |\tilde{\tau}_1 - \tau_1^*| \Bigg).
\end{aligned}$$

Thus, under $\bigcap_{i=0}^{7} \mathcal{E}_i$

$$|\widehat{V} - \widehat{V}^*| \leqslant C_L \left(\sqrt{\frac{(s_1 \vee s_2)\log(p \vee Kn)}{Kn}} + \frac{s_1^{3/2}\log^2(p \vee n)}{n} \right).$$

Then,

$$\begin{aligned}
\left|\widehat{V}^* - V^*\right| \leqslant &\left| \frac{1}{Kn}\sum_{j=1}^{m}\sum_{i=1}^{n}\left\{\frac{T_{ki}}{\pi(X_{ki}^T\theta^*)^2}\left(Y_{ki} - X_{ki}^T\beta^*\right)^2\right\} - \mathbb{E}\left(\frac{1}{\pi(X_{ki}^T\theta^*)}(Y_{ki} - X_{ki}^T\beta^*)^2\right) \right| \\
&+ \left| \frac{1}{Kn}\sum_{j=1}^{m}\sum_{i=1}^{n}\left\{\left(X_{ki}^T\beta^* - \tau_1^*\right)^2\right\} - \mathbb{E}\left(\left(X_{ki}^T\beta - \tau_1^*\right)^2\right) \right|.
\end{aligned}$$

Under $\mathcal{E}_6$:

$$\left|\widehat{V}^* - V^*\right| \leqslant C_L \sqrt{\frac{\log(p \vee Kn)}{Kn}}.$$

Under the events above, since V^* is bounded and positive:

$$\left|\sqrt{\frac{\widehat{V}}{V^*}} - 1\right| \leqslant \left|\frac{\widehat{V} - V^*}{V^* + \sqrt{\widehat{V}V^*}}\right| \leqslant C_L \left(\sqrt{\frac{(s_1 \vee s_2)\log(p \vee Kn)}{Kn}} + \frac{s_1^{3/2}\log^2(p \vee n)}{n} \right).$$

□

3.3 Misspecified models

Proposition 6. *Under Assumptions 1-6, with θ^* replaced by θ^o, the proposed estimator satisfies*

$$|\tilde{\tau}_1 - \hat{\tau}_{1,ps}^o| \leqslant C_L \left(\frac{\sqrt{s_2(s_1 \vee s_2)}\log(p \vee Kn)}{Kn} + \frac{s_1\sqrt{s_1 s_2 \log(p \vee Kn)\log^4(p \vee n)}}{n\sqrt{Kn}} \right)$$

with probability at least $1 - \frac{M}{n^8}$, where C_L is a sufficiently large constant and M is another constant depending on C_L.

Proof. The proof is an analog of theorem 4, where auxiliary lemmas can be established in a likewise manner. □

Lemma 7. *Under Assumptions 1-6, with β^* replaced by β^o, the proposed estimator satisfies*

$$|\tilde{\tau}_1 - \hat{\tau}^o_{1,om}| \leqslant C_L \left(\frac{\sqrt{s_2(s_1 \vee s_2)}\log(p \vee Kn)}{Kn} + \frac{s_1\sqrt{s_1 s_2 \log(p \vee Kn)\log^4(p \vee n)}}{n\sqrt{Kn}} \right)$$

with probability at least $1 - \frac{M}{n^8}$, where C_L is a sufficiently large constant and M is another constant depending on C_L.

Proof. The proof is an analog of theorem 4, where auxiliary lemmas can be established in a likewise manner. □

3.4 Initial estimator

Lemma 8. *For $\bar{\theta} = \bar{\theta}_K$, we have:*

$$\left\|\bar{\theta} - \theta^*\right\|_2 \leqslant C_1\sqrt{\frac{s_1 \log(p \vee n)}{n}}$$

holds with probability at least $1 - \frac{M}{(p \vee n)^8}$, where $J = K_1$, K_2, or K_3.

Proof. By lemma 10, we can see that event defined as:

$$\mathcal{E}_0 = \left\{ Q_1(\theta^* + \delta) - Q_1(\theta^*) - \nabla Q_1(\theta^*)^T\delta \geqslant \mu\left\|\delta\right\|_2^2 - \mu'\sqrt{\frac{\log p}{n}}\left\|\delta\right\|_2\left\|\delta\right\|_1 \right\}.$$

will hold with probability at least $1 - \frac{M}{(p \vee n)^8}$.

Then, under $\mathcal{E}_0$, we can apply corollary 1 of Negahban et al. (2012). Then, it shall be obtained that:

$$\left\|\bar{\theta}_K - \theta^*\right\|_2 \leqslant 3\frac{\sqrt{s_1}\lambda_{\text{ps, ini}}}{C},$$

where

$$\lambda_{\text{ps, ini}} \geqslant \left\|\nabla Q_1(\theta^*)\right\|_\infty.$$

For $\nabla Q_1(\theta^*)$, we have:

$$\nabla Q_1(\theta^*) = \frac{1}{n}\sum_{i=1}^{n}(1 - T_{i1})X_{i1S}^T - T_{i1}\exp\left(-X_{i1}^T\theta^*\right)X_{i1S}^T.$$

Since $\mathbb{E}(\nabla Q_1(\theta^*)) = 0$ and $T_{i1}, (1 - T_{i1})$ are bounded, with X_{i1S} being sub-Gaussian, by union bound and lemma 12,

$$\left\|\nabla Q_1(\theta^*)\right\|_\infty \leqslant \sqrt{\frac{\log(p \vee n)}{n}}.$$

Thus, we have:

$$\left\|\bar{\theta} - \theta^*\right\|_2 \leqslant C_1\sqrt{\frac{s_1 \log(p \vee n)}{n}}.$$

□

3.5 Restricted strong convexity(RSC) conditions

Lemma 9. *Under assumptions, we can see that the event defined as:*

$$\sum_{i=1}^{n} T_{ki} \geqslant c_1 n + C_L\sqrt{n\log n}$$

will hold with probability at least $1 - \frac{M}{(p \vee n)^8}$ for some constant c_1, C_L.

Proof. Let Z_{ki} be binomial random variables with probability c_0, where c_0 is the constant defined in 2. Then, we clearly have

$$\mathbb{P}\left(\left\{\sum_{i=1}^{n} T_{ki} \geqslant c_1 n + C_L\sqrt{n\log n}\right\}\right) \geqslant \mathbb{P}\left(\left\{\sum_{i=1}^{n} Z_{ki} \geqslant c_1 n + C_L\sqrt{n\log(p\vee n)}\right\}\right).$$

Clearly, Z_{ki} are sub-Gaussian random variables. Then, by lemma 13, the claimed bound can be obtained. □

Lemma 10. *The loss function of propensity score model follows restricted strong convexity with probability at least* $1 - \frac{M}{(p\vee n)^8}$*, where* M *is some positive constant. That is: for all* δ *s.t.* $\|\delta\|_2 \leqslant 1$

$$Q_1(\theta^* + \delta) - Q_1(\theta^*) - \nabla Q_1(\theta^*)^T\delta \geqslant \mu\|\delta\|_2^2 - \mu'\frac{\log p}{n}\|\delta\|_1^2,$$

where μ, μ' *is some positive constant.*

Proof. Under lemma 9, the claim is a result of Proposition 2 in Negahban et al. (2009). As demonstrated in Negahban et al. (2009), Assumption 3, outlined below, is integral to the proof of Lemma 10. Serving as a foundational assumption for high-dimensional data analysis, this condition ensures that the loss function maintains sufficient convexity even in high-dimensional spaces where traditional convexity might not universally apply.

Assumption 3 (Design)**.** *The minimal and maximal eigenvalues of* $\mathbb{E}[X_{ki}X_{ki}^T]$ *are contained in a bounded interval that does not contain zero.*

□

3.6 Concentration results

Lemma 11. *Consider* $\sum_{i=1}^{n} X_i$*, where* X_i *are zero-mean, independent sub-exponential random variables with parameter* $\alpha = a_i, \nu = \nu_i$*. Then,* $Y = \sum_{i=1}^{n} X_i$ *is a sub-exponential random variable with parameter* $\alpha = \max_i a_i, \nu = \sqrt{\sum_{i=1}^{n}\nu_i^2}$*.*

Lemma 12 (Bernstein Inequality for sub-exponential sums)**.** *Consider* $\sum_{i=1}^{n} X_i$*, where* X_i *are zero-mean, independent sub-exponential random variables with parameter* $\alpha = a_i, \nu = \nu_i$*. Let* $\alpha = \max_i a_i, \nu = \sqrt{\sum_{i=1}^{n}\nu_i^2}$*, we then notice that*

$$\mathbb{P}\left(\left|\frac{1}{n}\sum_{i=1}^{n} X_i\right| \geqslant t\right) \leqslant \begin{cases} \exp(-\frac{nt^2}{2\nu^2/n}) & \text{for } 0 \leqslant t \leqslant \frac{\nu^2}{n\alpha}, \\ \exp(-\frac{nt}{2\alpha}) & \text{for } t > \frac{\nu^2}{n\alpha}. \end{cases}$$

Lemma 13. *Consider* $\sum_{j=1}^{m}\sum_{i=1}^{n} X_{ki}$*, where* X_{ki} *are independent sub-exponential random variables with parameter* $\alpha = a_{ki}, \nu = \nu_{ki}$ *and common expectation* $E(X_{ki})$*, then the event*

$$\left|\frac{1}{Kn}\sum_{j=1}^{m}\sum_{i=1}^{n} X_{ki} - \mathbb{E}(X_{ki})\right| \geqslant C_L\sqrt{\frac{\log Kn}{Kn}}$$

will hold with probability at most $\frac{M}{(Kn)^8}$*, where* C_L *is some sufficiently large constant and* M *depends on* C_L*.*

The proof is a direct application of lemma 12 and union bound.

Lemma 14 (Rosenthal (1970), Theorem 3)**.** *Suppose that* $\{X_i\}_{i=1}^{n}$ *are zero-mean and independent random variables. For any* $p \geqslant 1$*, there exists a constant* R_p *that for any* $p \in \mathbb{N}$*:*

$$\mathbb{E}\left(\left(\sum_{i=1}^{n} X_i\right)^{2p}\right) \leqslant R_p\left(\sum_{i=1}^{n}\mathbb{E}(X_i^{2p}) + \left(\sum_{i=1}^{n}\mathbb{E}(X_i^2)\right)^p\right).$$

Lemma 15 (Tail bounds under moment conditions)**.** *Suppose that* $\{X_i\}_{i=1}^{n}$ *are zero-mean and independent random variables such that, for some fixed integer* $p \geqslant 1$*, they satisfy the moment bound* $\|X_i\|_{P,2p} \leqslant C_p$*. Then*

$$P\left(\left|\frac{1}{n}\sum_{i=1}^{n} X_i\right| \geqslant \delta\right) \leqslant B_p\left(\frac{1}{\sqrt{n}\delta}\right)^{2p} \qquad \text{for all}\delta > 0.$$

Proof. This lemma is a simple application of lemma 14. $\square$

Lemma 16. *Under the assumptions 3 and 4,*

1.

$$\frac{1}{Kn}\sum_{j=1}^{m}\sum_{i=1}^{n}(X_{ki}^T(\hat{\theta}-\theta^*))^2 \leqslant C\|\hat{\theta}-\theta^*\|_2^2,$$

2.

$$\left\|\hat{\beta}-\beta^*\right\|_1 \leqslant (s_1 \vee s_2)\sqrt{\frac{\log(p \vee n)}{n}},$$
$$\frac{1}{Kn}\sum_{j=1}^{m}\sum_{i=1}^{n}(X_{ki}^T(\hat{\beta}-\beta^*))^2 \leqslant \frac{(s_1 \vee s_2)\log(p \vee n)}{n}$$

with probability at least $1-\frac{M}{n^8}$, *where* C *is some sufficiently large constant, and* $\hat{\theta}, \hat{\beta}$ *are regularized PS, OR estimator, respectively.*

Proof. For the first claim, it is a result of lemma 6 and lemma 9 of Bradic et al. (2019), while for the second claim, it is a result of lemma S4 of Ning et al. (2020). $\square$

3.7 Bias without transferring Hessians

We are providing more details on the scenario where covariate shift exists but only first-order gradients are transferred. In particular, an additional bias term is introduced when only first-order gradients are transferred, rather than Hessians. Please find the detailed explanation below:

From Theorem 5 of Jordan et al. (2018), the error bound is determined by

$$\left\|\widetilde{\theta}-\theta^*\right\|_2 \leqslant C\left(\sqrt{\frac{s_1\log(p \vee Kn)}{Kn}}+\sqrt{s_1}\left\|\nabla^2 L_1(\boldsymbol{\theta}^*)-\nabla^2 L_N(\boldsymbol{\theta}^*)\right\|_\infty \left\|\bar{\boldsymbol{\theta}}-\boldsymbol{\theta}^*\right\|_1\right),$$

where $\bar{\boldsymbol{\theta}}$ is the initial estimator. If Lasso is applied to the local data to obtain initial $\bar{\boldsymbol{\theta}}$, then $\left\|\bar{\boldsymbol{\theta}}-\boldsymbol{\theta}^*\right\|_1 \leqslant Cs_1\sqrt{\frac{\log p \vee n}{n}}$ holds.

- ***If there does not exist covariate shift***, we are assuming $\mathbb{E}(\nabla^2 L_1(\boldsymbol{\theta}^*)) = \mathbb{E}(\nabla^2 L_k(\boldsymbol{\theta}^*))$ for all $k \in \{1,\ldots,K\}$, then we have

  $$\left\|\nabla^2 L_1(\boldsymbol{\theta}^*)-\nabla^2 L_N(\boldsymbol{\theta}^*)\right\|_\infty \leqslant \sqrt{\frac{\log(p \vee n)}{n}}.$$

  Following Jordan et al's idea, the error bound is

  $$\left\|\widetilde{\theta}-\theta^*\right\|_2 \leqslant C\left(\sqrt{\frac{s_1\log(p \vee Kn)}{Kn}}+s_1^{3/2}\frac{\log(p \vee n)}{n}\right),$$

  This error bound is better than the classical Lasso l_2-error bound $\sqrt{\frac{s_1\log(p \vee n)}{n}}$, which only uses the local data from a single local site.

- However, ***if there exists covariate shift***, i.e. $\mathbb{E}(\nabla^2 L_1(\boldsymbol{\theta}^*)) \neq \mathbb{E}(\nabla^2 L_k(\boldsymbol{\theta}^*))$ for some $k \in \{1,\ldots,K\}$, we have

  $$\left\|\nabla^2 L_1(\boldsymbol{\theta}^*)-\nabla^2 L_N(\boldsymbol{\theta}^*)\right\|_\infty = O(1).$$

  If we still follow Jordan et al's idea by only transferring first-order gradients, the error bound becomes

  $$\left\|\widetilde{\theta}-\theta^*\right\|_2 \leqslant C\left(\sqrt{\frac{s_1\log(p \vee Kn)}{Kn}}+s_1^{3/2}\sqrt{\frac{\log(p \vee n)}{n}}\right),$$

  This error bound is worse than the classical Lasso l_2-error bound where only local data is used.

Therefore, when there exits covariate shift between different sites, with the aim of improving the convergence rate, we require not only transferring the first-order gradients but also the Hessians to obtain the improved rate

$$\left\|\tilde{\theta}-\theta^*\right\|_2 \leqslant C_L\left(\sqrt{\frac{s_1\log(p\vee Kn)}{Kn}}+\frac{s_1^{3/2}\log^2(p\vee n)}{n}\right).$$

4 Additional simulations

In this section, to provide a better understanding of the methodology, we conduct additional numerical simulations. We compare the performance of the proposed method from two perspectives: splitting data versus not splitting it, and the comparison between splitting patient-level data and splitting sites. Without loss of generality, for $k=1,\ldots,K$ and $i=1,\ldots,n$, the treatment T_{ki} are generated from a logistic regression with $\pi_{ki}=\text{expit}(-0.5+0.5X_{ki1}+0.3X_{ki2}-0.3X_{ki3}+0.3X_{ki4}-0.3X_{ki5})$, the potential outcomes satisfy $Y_{ki}(1)=2+0.3X_{ki1}+0.2X_{ki2}-0.2X_{ki3}+0.2X_{ki4}-0.2X_{ki5}+\epsilon_{ki1}$ and $Y_{ki}(0)=1+0.3X_{ki1}+0.2X_{ki2}-0.2X_{ki3}+0.2X_{ki4}-0.2X_{ki5}+\epsilon_{ki0}$, where ϵ_{ki1} and ϵ_{ki0} are i.i.d from $N(0,1)$, while the p-dimensional covariates are generated from $\mathbf{X}_{ki}\sim N(0,\mathbf{\Sigma}_k)$. For simplicity, we only consider the heterogeneous case (II) in the paper, specifically

(II) **Heterogeneous covariates (i.e., covariate shift) with** $p<n$: We consider the dimension with $p=100$ and the sample size in each site is fixed at $n=200$, while the covariance matrix $\mathbf{\Sigma}_k$ is set to be $\Sigma_{k;st}=\rho_k^{|s-t|}$, where $\rho_k\sim\text{Uniform}(0.2,0.8)$ for $k=1,\ldots,K$. In this case, the simple size is larger than the dimension, and there is a shift in the distribution of covariates across sites.

The comparison results of splitting data versus not splitting it are depicted in Figure 1, where DisC^{2}o-HD-1 and DisC^{2}o-HD-2 represent our proposed approaches involving the splitting of K, while DisC^{2}o-HD-1-WS and DisC^{2}o-HD-2-WS denote the corresponding methods without splitting any data. We can see that the performance of DisC^{2}o-HD-2 are close to DisC^{2}o-HD-2-WS when K is large, while DisC^{2}o-HD-1 can outperform DisC^{2}o-HD-1-WS when K is large. In summary, while we employ data splitting for proof convenience, in numerical analysis, it's also feasible to apply the proposed method without splitting any data.

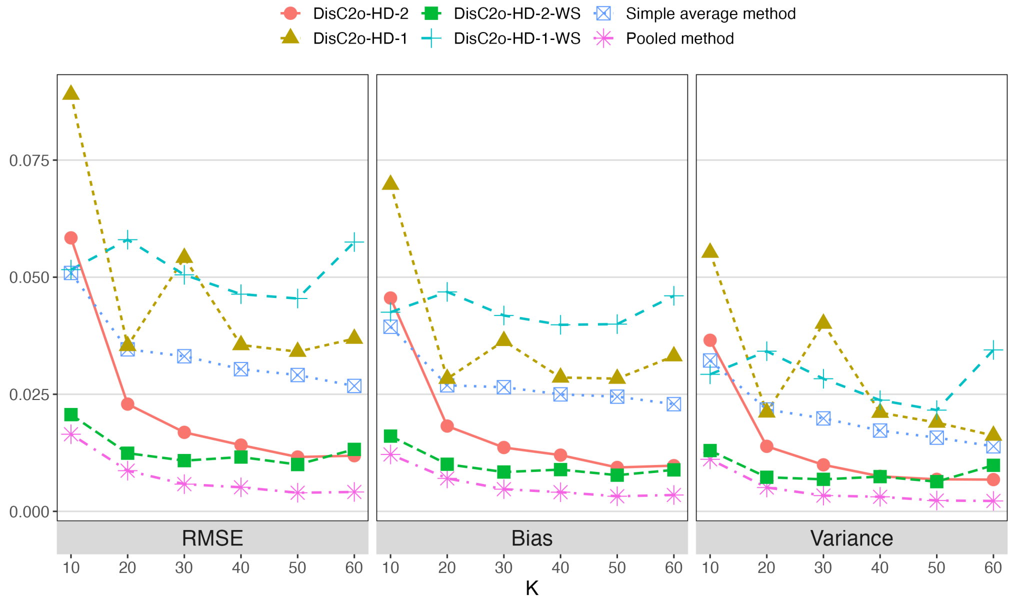

Figure 1: Comparison results of different methods under scenario (II) – heterogeneous covariates with $p<n$

In the comparison between splitting patient-level data and splitting sites scenario, we fixed $K=15$ and compare the ATE estimation error by repeating the process 100 times. The DisC^{2}o-HD-1 and DisC^{2}o-HD-2 are our proposed approaches involving splitting K, while the DisC^{2}o-HD-1-SN and DisC^{2}o-HD-2-SN approaches involve splitting patient-level data n.

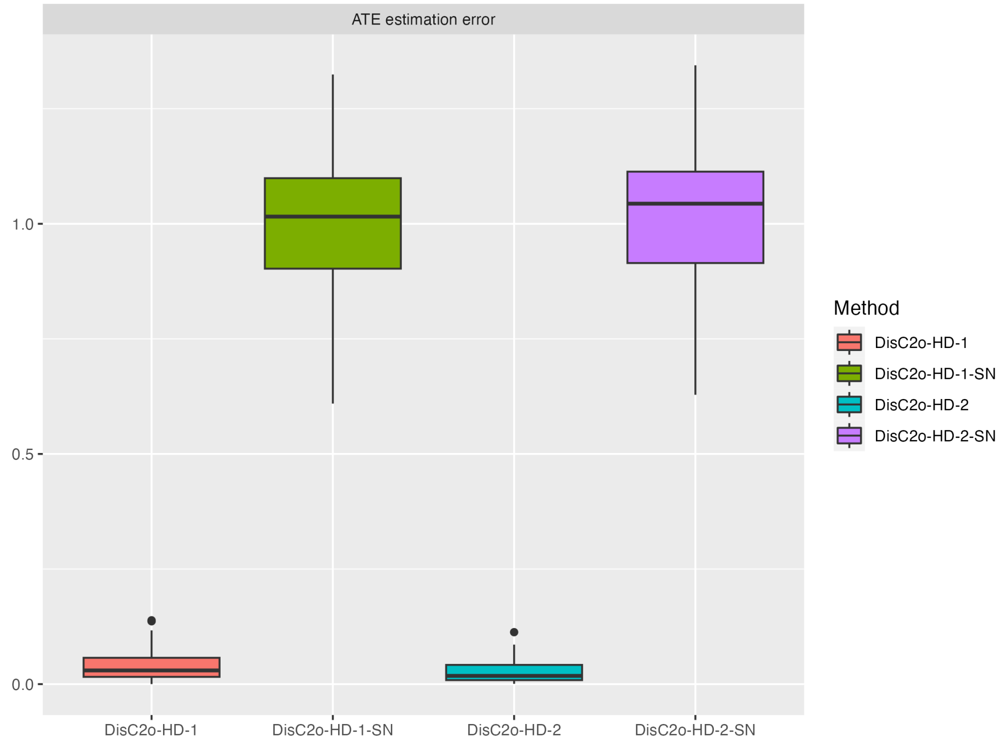

Figure 2: Comparison results of different methods under scenario (II) – heterogeneous covariates with $p < n$

As depicted in Figure 2, it is evident that the DisC^{2}o-HD-1 and DisC^{2}o-HD-2 methods exhibit greater robustness and yield smaller ATE estimation errors compared to the DisC^{2}o-HD-1-SN and DisC^{2}o-HD-2-SN methods, thereby supporting our decision to split K.

References

Bradic, J., Wager, S., and Zhu, Y. (2019). Sparsity double robust inference of average treatment effects. *arXiv preprint arXiv:1905.00744*.

Jordan, M. I., Lee, J. D., and Yang, Y. (2018). Communication-efficient distributed statistical inference. *Journal of the American Statistical Association*.

Negahban, S., Yu, B., Wainwright, M. J., and Ravikumar, P. (2009). A unified framework for high-dimensional analysis of m-estimators with decomposable regularizers. *Advances in neural information processing systems*, 22.

Negahban, S. N., Ravikumar, P., Wainwright, M. J., and Yu, B. (2012). A unified framework for high-dimensional analysis of m-estimators with decomposable regularizers. *Statistical science*, 27(4):538–557.

Ning, Y. and Liu, H. (2017). A general theory of hypothesis tests and confidence regions for sparse high dimensional models. *The Annals of Statistics*, 45(1):158–195.

Ning, Y., Sida, P., and Imai, K. (2020). Robust estimation of causal effects via a high-dimensional covariate balancing propensity score. *Biometrika*, 107(3):533–554.

Rosenbaum, P. R. and Rubin, D. B. (1983). The central role of the propensity score in observational studies for causal effects. *Biometrika*, 70(1):41–55.

Rosenthal, H. P. (1970). On the subspaces of $\mathcal{L}^p(p > 2)$ spanned by sequences of independent random variables. *Israel Journal of Mathematics*, 8(3):273–303.

Tan, Z. (2020). Model-assisted inference for treatment effects using regularized calibrated estimation with high-dimensional data. *The Annals of Statistics*, 48(2):811–837.

Van de Geer, S., Bühlmann, P., Ritov, Y., and Dezeure, R. (2014). On asymptotically optimal confidence regions and tests for high-dimensional models. *The Annals of Statistics*, 42(3):1166–1202.
